# Cytosolic DNA Sensors and CNS Responses to Viral Pathogens

**DOI:** 10.3389/fcimb.2020.576263

**Published:** 2020-09-16

**Authors:** Austin M. Jeffries, Ian Marriott

**Affiliations:** Department of Biological Sciences, The University of North Carolina at Charlotte, Charlotte, NC, United States

**Keywords:** DNA sensors, astrocytes, microglia, neuroinflammation, viral encephalitis

## Abstract

Viral central nervous system (CNS) infections can lead to life threatening encephalitis and long-term neurological deficits in survivors. Resident CNS cell types, such as astrocytes and microglia, are known to produce key inflammatory and antiviral mediators following infection with neurotropic DNA viruses. However, the mechanisms by which glia mediate such responses remain poorly understood. Recently, a class of intracellular pattern recognition receptors (PRRs), collectively known as DNA sensors, have been identified in both leukocytic and non-leukocytic cell types. The ability of such DNA sensors to initiate immune mediator production and contribute to infection resolution in the periphery is increasingly recognized, but our understanding of their role in the CNS remains limited at best. In this review, we describe the evidence for the expression and functionality of DNA sensors in resident brain cells, with a focus on their role in neurotropic virus infections. The available data indicate that glia and neurons can constitutively express, and/or can be induced to express, various disparate DNA sensing molecules previously described in peripheral cell types. Furthermore, multiple lines of investigation suggest that these sensors are functional in resident CNS cells and are required for innate immune responses to viral infections. However, it is less clear whether DNA sensormediated glial responses are beneficial or detrimental, and the answer to this question appears to dependent on the context of the infection with regard to the identity of the pathogen, host cell type, and host species. Defining such parameters will be essential if we are to successfully target these molecules to limit damaging inflammation while allowing beneficial host responses to improve patient outcomes.

## Introduction

Infection of the central nervous system (CNS) can result in encephalitis, a condition that is characterized by severe neuroinflammation resulting in fever, headaches, altered consciousness, seizures, and even death (Roos, 1999). Between 2000 and 2010 there were 7.3 encephalitis cases per 100,000, with most identified etiologies (48.2%) being attributable to viral infections (George et al., 2014). Since the mechanisms that lead to CNS inflammation following infection are poorly understood, current treatment strategies include general immune suppression, and/or antiviral therapy (Chaudhuri and Kennedy, 2002; George et al., 2014; Venkatesan and Geocadin, 2014). Traditionally, it was thought that infiltrating peripheral monocytes and leukocytes were the major contributors of pro-inflammatory mediator production in encephalitis as most resident CNS cells were assumed to lack immune functions. However, it is now appreciated that glial cells, most notably microglia and astrocytes, play a critical role in immune surveillance in the CNS and are important contributors to both protective and detrimental host responses to infectious agents (Aloisi, 2000, 2001; Bsibsi et al., 2002, 2006; Bowman et al., 2003; Furr et al., 2008, 2011; Chauhan et al., 2009; Liu et al., 2010; Serramía et al., 2015).

Glial cells can produce an array of proinflammatory and antiviral mediators following infection (Chauhan et al., 2009; Furr and Marriott, 2012) and it is now known that they accomplish this via members of multiple families of pattern recognition receptors (PRRs). These PRRs recognize numerous pathogen-associated molecular patterns (PAMPs) and/or damage associated molecular patterns (DAMPs) and trigger transcription factor activation that, in turn, elicits proinflammatory and antiviral mediator production. Of these, the most widely and best studied glial PRRs are the cell surface and endosomal Toll-like receptors (TLRs) and the cytosolic nucleotide-binding and oligomerization domain (NOD)-like receptors (NLRs) (Sterka et al., 2006; Chauhan et al., 2009; Rebsamen et al., 2009; Liu et al., 2010; Dai et al., 2014; Reinert et al., 2016; Su and Zheng, 2017). More recently, multiple classes of cytosolic nucleic acid sensors have been discovered that are likely have an important function during active viral infections due to their intracellular location. These include RNA sensors, such as the retinoic acid-inducible gene-I (RIG-I)-like receptor (RLR) family, and the possible roles and importance of these molecules in glial immune responses have been discussed in depth elsewhere (Furr et al., 2008; Furr and Marriott, 2012; Carty et al., 2014; Nair and Diamond, 2015; Zohaib et al., 2016). However, the importance of DNA sensors, including cyclic guanosine monophosphate-adenosine monophosphate (cGAMP) synthase (cGAS), in viral CNS infections and the initiation of glial immune functions remains more controversial, despite evidence for their antiviral function in peripheral lymphoid and myeloid cells (Unterholzner, 2013; Cai et al., 2014; Dhanwani et al., 2018; Lugrin and Martinon, 2018). In this review article, we will discuss the evidence for the expression and function of DNA sensors in resident CNS cells, their role during viral infections, and their potential as targets for therapeutic intervention.

## cGAS/Sting

Perhaps the most well-known and best studied cytosolic DNA sensor is cGAS. This molecule directly binds to double stranded DNA and then catalyzes the production of the secondary messenger, 2′3′cyclic GMP-AMP (cGAMP) (Gao P. et al., 2013; Sun et al., 2013; Zhang X. et al., 2013). This secondary messenger subsequently binds to the downstream adaptor protein stimulator of interferon genes (STING), which initiates the phosphorylation of tank binding kinase 1 (TBK1), and interferon regulatory factor 3 (IRF3), and activates nuclear factor kappalight-chain-enhancer of activated B cells (NF-kB). Such transcription factor activation precipitates the expression of interferon-beta (IFN-β) and other antiviral and/or pro-inflammatory cytokines (Ishikawa et al., 2009; Li et al., 2013; Abe and Barber, 2014; Fang et al., 2017; Sun et al., 2017; Aarreberg et al., 2019). Since its discovery in 2013, cGAS has been demonstrated to play a critical role in recognizing and eliminating a diverse array of pathogens, either through direct recognition of microbial DNA or indirect recognition of retroviral DNA intermediates or damage associate molecular patterns (DAMPS), such as released mitochondrial DNA (Gao D. et al., 2013; Abe and Barber, 2014; Dai et al., 2014; Herzner et al., 2015; Paijo et al., 2016; Fang et al., 2017; Sun et al., 2017; Cheng et al., 2018; Wong et al., 2019). As discussed elsewhere (Cai et al., 2014; Chen et al., 2016; Dhanwani et al., 2018; Ablasser and Chen, 2019), numerous studies have demonstrated the expression and function of cGAS in peripheral leukocytic and non-leukocytic cell types, such as human plasmacytoid dendritic cells, macrophages, monocytes, helper T-lymphocytes, and endothelial cells (as summarized in Table 1). However, less attention has been given to the role of this sensor in the CNS and the immune responses of glial cells (Lahaye et al., 2013; Li et al., 2013; Dai et al., 2014; Ma Z. et al., 2015; West et al., 2015; Bode et al., 2016; Paijo et al., 2016; Vermeire et al., 2016; Luecke et al., 2017; Su and Zheng, 2017; Sun et al., 2017; Swanson et al., 2017).

**Table 1:** Expression and antiviral activity of intracellular DNA sensors in peripheral cell types.

**Sensor**	**Cell type**	**Ligand**	**Antiviral activity**	**Recognized pathogens**	**References**
ZBP1	Mouse primary fibroblast, liver macrophages, BMDM Mouse cell lines, L929, SVEC4-10, NIH3T3, 3T3-SA Human cell lines HT-29, A549, HepG2	dsDNA or RNA	IFN expression NF-κB activation, cell death	HSV-1, IAV, CMV, vaccinia virus, ZIKV, WNV	Upton et al., 2012; Pham et al., 2013; Kuriakose et al., 2016; Lin et al., 2016; Maelfait et al., 2017; Guo et al., 2018; Daniels et al., 2019; Ingram et al., 2019; Rothan et al., 2019; Yang et al., 2020
cGAS	Mouse primary lung fibroblasts, GM-CSF DC, FLt3L DC, BMDM Murine cell lines, L929, RAW 264.7 Hamster cell line BHK Primary human PBMCs, MDM, MDDC, plasmacytoid dendritic cells, PBMCs, CD4+ T cells Human cell lines, THP1, HFFs, A549, HUVEC, EA.hy926	dsDNA	IFN expression, inflammasome priming	HIV, HSV, MLV, SIV, HCMV, DENV, ectromelia virus	Gao D. et al., 2013; Li et al., 2013; Sun et al., 2013; Herzner et al., 2015; Ma F. et al., 2015; Bode et al., 2016; Vermeire et al., 2016
IFI16	Mouse primary corneal epithelium Mouse cell line RAW 264.7 Primary human MDM, keratinocytes, PBMCs, CD4+ T cells, hepatocytes Human cell lines, THP1, hTCEpi, HFFs, BJAB, HMVEC, BCBL1, 184B5, HCC1937, TIME, HELF, U2OS, HUVEC, Akata cells, MUTU1, huh7, HepG2, HeLa, HepaRG cells, C666-1, Raji, LCL	dsDNA	Inflammasome activation, IFN-β production, transcriptional regulation	HSV-1, KSHV, HIV, EBV, HBV	Unterholzner et al., 2010; Conrady et al., 2012; Orzalli et al., 2012; Ansari et al., 2013; Cuchet-Lourenco et al., 2013; Jakobsen et al., 2013; Dell'Oste et al., 2014; Diner et al., 2015; Dutta et al., 2015; Iqbal et al., 2016; Pisano et al., 2017; Lum et al., 2019; Roy et al., 2019; Yang et al., 2020
AIM2	Primary mouse BMDM, BMDC, MEF, alveolar macrophages, peritoneal macrophages Mouse cell lines NR9456, B6-MCL Primary human dermal fibroblasts, keratinocytes Human cell lines, THP1, ATII	dsDNA	Inflammasome activation	HPV, HBV, HCMV	Fernandes-Alnemri et al., 2009; Hornung et al., 2009; Rathinam et al., 2010; Reinholz et al., 2013; Sagulenko et al., 2013; Ekchariyawat et al., 2015; Corrales et al., 2016; Gray et al., 2016; Huang et al., 2017; Nakaya et al., 2017; Zhang et al., 2017; Chen et al., 2018
DDX41	Primary mouse BMDC, peritoneal macrophages, MEF Mouse cell lines, D2SC, L929 Primary human PBMCs Human cell line THP1	dsDNA or DNA:RNA hybrid	IFN and ISG expression	HSV-1, adenovirus, MLV, IAV (mitochondrial DNA)	Zhang Z. et al., 2011, 2013; Lee et al., 2015; Stavrou et al., 2015; Moriyama et al., 2019
DNA-PK	Primary mouse MEF Primary human monocytes, MDDC Human cell lines, HEK293, HeLa, THP1, SK-hep-1, HepG2.2.15, U937s, HFFs	dsDNA	IFN expression	HSV-2, HTLV, HBV, vaccinia virus	Zhang X. et al., 2011; Ferguson et al., 2012; Peters et al., 2013; Li et al., 2016; Wang et al., 2017; Scutts et al., 2018
RNA-pol III	Primary mouse MEF, dendritic cells Mouse cell line RAW264.7 Primary human PBMCs, MDDC Human cell line HEK293	RNA	IFN expression NF-κB activation	adenovirus, HSV-1, EBV	Ablasser et al., 2009; Chiu et al., 2009

The first description of cGAS expression in resident CNS cell types came from Cox et al. (2015), who established the constitutive and inducible expression of mRNA encoding cGAS in murine microglia. Interestingly, while they observed neither constitutive nor IFN-β-inducible expression of cGAS in murine astrocytes, siRNA-mediated cGAS knockdown decreased IFN-β activity in both microglia and astrocytes following exposure to exogenous dsDNA (Cox et al., 2015). Further support for the presence of cGAS in glia has since been provided by our demonstration that primary human microglia and astrocytes both constitutively express cGAS protein and its downstream adaptor molecule STING (Jeffries and Marriott, 2017).

Circumstantial evidence for the functional importance of cGAS in the brain lays in the recognized ability of many important CNS pathogens to interfere with this sensor and/or it's signaling pathway (as summarized in Table 2). For example, herpes simplex virus 1 (HSV-1), the dsDNA virus that is the most common cause of fatal sporadic encephalitis, has multiple gene products that can interfere with the cGAS-STING signaling pathway (Bradshaw and Venkatesan, 2016). The HSV-1 encoded protein UL37 attenuates the enzymatic activity of cGAS and lowers cGAMP production in human monocytes and fibroblasts, thereby decreasing antiviral gene expression (Zhang et al., 2018). Mutations in UL37 that interfere with its deamidase activity prevent this protein from inhibiting cGAS and lead to lower HSV-1 titers in the brain following infection (Zhang et al., 2018). Interestingly, such an inhibitory activity appears to show species specificity as UL37 molecule does not appear to be important in infections in some non-human primate species (Zhang et al., 2018). Another HSV-1 product, UL41 (virion host shutoff protein), has been shown to decrease IFN-β production in a human epithelial cell line expression system by reducing cGAS protein expression (Su and Zheng, 2017), and its importance in disrupting cGAS-mediated antiviral responses has been illustrated by the ability of cGAS knockdown to increase viral production of a UL41 null mutant HSV-1 strain, but not a wild type strain (Su and Zheng, 2017). Furthermore, additional HSV-1 products have been demonstrated to target down-stream signaling molecules in the cGAS STING pathway. For instance, UL36 can prevent NF-κB activation by cleaving IκBa poly ubiquitin chains while UL46 and ICP27 interact with STING and TBK1 to prevent the activation of IRF3 and interferon stimulated gene (ISG) expression (Christensen et al., 2016; Deschamps and Kalamvoki, 2017; Ye et al., 2017; You et al., 2019). Since all members of the family Herpesviridae appear to target cGAS [Table 2 and as reviewed in Chan and Gack (2016) and Phelan et al. (2020)], and such viruses can cause latent infections, it is tempting to speculate that the inhibition of this sensor may play a critical role in establishing viral latency. Such a mechanism could be of particular importance for HSV encephalitis as the reactivation of a latent infection is thought to be a key contributor to the development of this condition (Menendez and Carr, 2017).

**Table 2:** Viral inhibitors of DNA sensing pathways.

**Sensor**	**Virus**	**Viral product**	**Inhibition mechanisms**	**References**
STING	Coronavirus	Papain-like proteases	Blocks dimerization and signaling	Sun et al., 2012
	DENV	NS2B3	Cleavage (human only)	Aguirre et al., 2012
	HCMV	UL122 (IE86)	Facilitated degradation	Kim et al., 2017
		US9	Blocks dimerization and association with TBK-1	Choi et al., 2018
		UL82	Inhibits translocation and impairs TBK1 and IRF3 recruitment	Fu et al., 2017
	HCV	NS4B	Suppresses accumulation and activation	Yi et al., 2016
	HSV-1	ICP27	Inhibits TBK1/STING signaling	Christensen et al., 2016
		γ_1_34.5	Disrupts translocation	Pan et al., 2018
cGAS	DENV	NS2B3	Autophagosomal cleavage/degradation	Aguirre et al., 2017
	EBV	KSHV ORF52 homolog	Inhibits activity	Wu et al., 2015
	HCMV	pUL83	Reduces cGAMP production	Biolatti et al., 2017
		UL31	Interferes with DNA binding	Huang Z. F. et al., 2018
	HSV-1	UL37	Inhibits cGAMP production (not in NHP)	Zhang et al., 2018
		VP22	Inhibiting enzymatic activity	Huang J. et al., 2018
	KSHV	ORF52	Inhibiting enzymatic activity	Wu et al., 2015
	MHV68	KSHV ORF52 homolog		
	RRV	KSHV ORF52 homolog		
cGAS/STING	HIV-2/SIV	VPX	Blocks cGAS/STING mediated NF-κB activation	Su et al., 2019
	HSV-1	UL41	Unknown	Su and Zheng, 2017
		UL24	Prevents NF-κB translocation	Xu et al., 2017
		UL36	Prevents NF-κB activation by cleaving IkBa polyubiquiting chains	Ye et al., 2017
		UL46	Interacts with TBK1 and STING to reduce ISG expression	Deschamps and Kalamvoki, 2017
		ICP27	Interacts with TBK1 and STING to prevent IRF3 activation	Christensen et al., 2016
	KSHV	ORF36	Unknown	Ma Z. et al., 2015
		ORF73		
		ORF57		
		ORF45		
		ORF55		
		vIRF1	Inhibits STING/TBK1 interactions	
	KSHV	cytoplasmic LANA	Binds cGAS and prevents TBK1 and IRF3 phosphorylation	Zhang G. et al., 2016
cGAS/STING/ RIG-I	HPV	SUV39H1	Represses transcription at their promoter regions	Lo Cigno et al., 2020
IFI16	HCMV	pUL97	Mislocalization	Dell'Oste et al., 2014
	HSV-1	ICP0	Increases degradation	Orzalli et al., 2012; Diner et al., 2015; Li et al., 2016
	KSHV	Unknown	Increases degradation	Roy et al., 2016
IFI16/STING	HSV-1	UL46	Reduces protein expression of each and interferes with STING/TBK1 interaction	Deschamps and Kalamvoki, 2017
ZBP1	HSV-1	ICP6	Blocks human RIP3/MLKL interactions (but activates RIPK3 in mice)	Wang et al., 2014; Huang et al., 2015; Guo et al., 2018
	MCMV	M45	Blocks ZBP1/RIP3 interactions	Upton et al., 2012
AIM2	HCMV	UL83	Prevents IL-1β maturation and may increase IFI16 degradation	Huang et al., 2017
		IE86	Blocks IL-1β secretion	Botto et al., 2019
		IE86	Inhibits NF-κB gene transcription and IL-1β release	
	HSV-1	VP22	Prevents inflammasome oligomerization	Maruzuru et al., 2018
DDX41	HSV-1	miR-H2-3p	Inhibits DDX41 transcription	Duan et al., 2019

In addition to DNA viruses, other neurotropic viruses, such as positive stranded RNA viruses from the family Flaviviridae, can also hamper cGAS activity (Table 2). For instance, Dengue virus (DENV) that can cause encephalitis has been shown to target cGAS for degradation and prevent it from detecting released mitochondrial DNA in human monocyte-derived dendritic cells and monocytic and fibroblastic cell lines (Aguirre et al., 2017; Sun et al., 2017).

Specifically, the DENV protein NS2B3 has been shown to directly interact with cGAS and target it for lysosomal degradation (Aguirre et al., 2017). In addition, this viral product can also cleave the downstream signaling molecule STING, further disrupting cGAS-mediated antiviral signaling. Interestingly, this viral product also shows species specificity similar to the HSV-1 product UL37 as NS2B3 targets human STING but does not cleave this molecule in mouse or non-human primate cells (Stabell et al., 2018). Similarly, Zika virus (ZIKV), which came to prominence for its role in CNS and peripheral nerve pathologies including microcephaly and Guillain-Barré syndrome, can cleave STING via its NS2B3 protein and this effect, again, is restricted to human rather than murine cells (Ding et al., 2018). ZIKV can further disrupt cGASSTING signaling by stabilizing caspase-1 protein. This results in cGAS cleavage and reduced expression of type I IFNs and ISGs, and the promotion of inflammasome activation and ZIKV production (Zheng et al., 2018). Importantly, Zheng et al. (2018) demonstrated that cGAS deficiency or caspase-1 inhibition leads to increased cellular levels of ZIKV genetic material. Such findings therefore support the therapeutic potential of augmenting cGAS-STING mediated responses to combat debilitating neurotropic RNA virus infections.

More direct evidence of the importance of cGAS-STING signaling during viral CNS infections comes from the study of HSV-1 infection in STING deficient animals. Parker et al. (2015) demonstrated that STING deficient animals show increased susceptibility to intracerebral HSV-1 infection, with all succumbing within 3–5 days of infection and little mortality in age-matched wildtype animals. Interestingly, it appears that this increased susceptibility is dependent on the route of infection, as STING deficient animals show survival rates comparable to wildtype mice following administration via the cornea, despite the presence of high viral titers in the cornea and trigeminal ganglion (Parker et al., 2015). This phenomenon was subsequently confirmed (Royer and Carr, 2016) and the apparent discrepancy in lethality is likely to be due to difference in the distribution of HSV-1 within the CNS. High viral titers are limited to the trigeminal ganglion following corneal HSV-1 infection, while intracerebral infection results in widely disseminated HSV-1 infection throughout the CNS (Parker et al., 2015). In addition, it should be noted that susceptibility to HSV-1 appears to be strain dependent as STING deficient animals succumb to neuroinvasive strains of HSV-1 following corneal infection more rapidly than wild type animals (Parker et al., 2015). Regardless, it is clear that STING plays a role in HSV-1 neuroinvasion and is critical for protective host responses once the virus has disseminated throughout the CNS (Parker et al., 2015; Royer and Carr, 2016). The increased susceptibility of STING deficient animals to HSV-1 neuroinvasion may be due, at least in part, to a decreased expression of the ISG tetherin [also known as bone marrow stromal antigen 2 (BST2)], as these animals exhibit decreased expression of this ISG (amongst others) during infection (Royer and Carr, 2016), and tetherin depletion has been shown to increase HSV1 titers in the trigeminal ganglion (Royer and Carr, 2016).

Consistent with these studies employing STING deficient animals, treatment with the STING agonist dimethlxanthenone-4-acetic acid (DMXAA) has been demonstrated to increase IFN-β expression by HSV-1 infected mouse fibroblasts and to lower the production of viral particles by these cells (Cerón et al., 2019). Importantly, *in vivo* DMXAA treatment can lower viral titers in the cornea, trigeminal ganglion, and brainstem, of mice infected with the neuroinvasive McKrae strain of HSV-1, and this is reflected by increased survival and improved neurological outcomes in these animals (Cerón et al., 2019). As such, these studies provide a tantalizing glimpse of the potential of targeting the cGAS-STING pathway to treat CNS infections.

While these studies illustrate the importance of cGAS-STING signaling in HSV-1 infections of the CNS, the specific role of this sensor system in glia was established by Reinert et al. (2016) in a mouse model of HSV-1 encephalitis. They demonstrated that cGAS deficiency resulted in a phenotype that matched that observed in STING deficient mice following ocular HSV-1 infection (Reinert et al., 2016). Furthermore, they established that microglia were the primary producers of IFN-β after HSV-1 challenge and showed that only this glial cell type produced higher viral titers *in vitro* following the loss of STING (Reinert et al., 2016). *In vivo*, however, neurons and astrocytes showed greater numbers of HSV-1 viral particles in STING deficient mice (Reinert et al., 2016). This discrepancy was explained by the observation that astrocytes and neurons initiate antiviral programs *in vivo* in response to IFN-β produced by microglia in a TLR3-dependent manner (Reinert et al., 2016). This suggests that microglia represent the first responders to HSV-1 infection in the CNS.

Consistent with these findings in mice, we have shown that cytosolic administration of a dsDNA ligand can phosphorylate IRF3 and induce IFN-β mRNA expression in primary human microglia and astrocytes (Jeffries and Marriott, 2017), and we have demonstrated that such responses are largely dependent on cGAS expression (Jeffries and Marriott, 2017; Jeffries et al., 2020). Furthermore, we showed that ISG expression is lower in cGAS deficient human microglia both at rest and following infection with HSV-1 (Jeffries et al., 2020). However, while cGAS can contribute to antiviral gene expression, the absence of cGAS had no effect on HSV-1 production in infected human microglia (Jeffries et al., 2020). Given the recognized ability of HSV-1 products to abrogate cGAS-STING signaling in peripheral human but not murine cell types, we assessed cGAS protein levels in human glial cells following HSV-1 challenge. We found that the expression of this sensor was markedly reduced in human microglial and astrocytic cells following infection (Jeffries et al., 2020), highlighting the ability of viruses such as HSV-1 to circumvent PRR-mediated immune responses. As such, improving the stability and/or expression of cGAS/STING signaling components might be a viable approach to combat viral infections of the CNS and/or periphery.

## IFI16

Interferon gamma inducible protein 16 (IFI16) is a member of the Pyrin and HIN domain (PHYIN) family of proteins that can serve as an intracellular DNA sensing molecule. PHYIN proteins are characterized by the presence of an N-terminal pyrin domain and one or two Cterminal HIN domains (Unterholzner et al., 2010). The HIN domains bind DNA while the pyrin domain is required for protein-protein interactions (Unterholzner et al., 2010). IFI16, and its mouse ortholog p204, was the first PHYIN family members demonstrated to induce IFN-β in response to transfected DNA (Unterholzner et al., 2010). Additionally, IFI16 has been shown to interact with STING and knockdown of either of these proteins leads to reduced IFN-β production by the human and mouse monocytic cell lines THP-1 and RAW 264.7, respectively (Cridland et al., 2012). Interestingly, similar findings were described in murine astrocytes and microglia where p204 knockdown was shown to reduce IFN-β expression following DNA transfection (Cox et al., 2015), and our own studies indicate that human microglial and astrocytic cells constitutively express robust levels of IFI16 protein (Jeffries et al., 2020).

However, IFI16 does not appear to contribute to IFN-β expression by human foreskin fibroblasts (HFF) stimulated with exogenous DNA (Orzalli et al., 2015). While this finding might be indicative of cell type-specific differences, our studies showing that IFI16 knockdown has no effect on IFN-β protein production by a human microglia cell line following BDNA transfection also indicate that this putative DNA sensor is not required for such responses (Jeffries et al., 2020). Furthermore, Gray et al. (2016) used a mouse model lacking all 13 PHYIN family members to demonstrate that these receptors were dispensable for IFN responses to DNA transfection in bone marrow derived macrophages and mouse embryonic fibroblasts. But despite an apparent lack of involvement in IFN production, this study did identify a requirement for PHYIN family members in inflammasome activation, as characterized by the maturation of the potent pro-inflammatory cytokines IL-1β and IL-18 (Gray et al., 2016). Such a role for IFI16 in linking DNA sensing and inflammasome activation is supported by multiple studies (Ansari et al., 2013, 2015; Johnson et al., 2013; Dutta et al., 2015; Iqbal et al., 2016; Orzalli et al., 2016) and is discussed in depth elsewhere (Dell'Oste et al., 2015), but it is currently unknown whether IFI16-mediated inflammasome activation occurs in CNS cell types.

While there is conflicting evidence for the role of IFI16 in IFN-β responses to foreign DNA challenge, multiple lines of investigation indicate that this DNA sensor contributes to IFN-β and ISG expression following viral infection (Orzalli et al., 2012; Jakobsen et al., 2013; Ansari et al., 2015; Diner et al., 2015; Ma F. et al., 2015; Li et al., 2016; Zhang D. et al., 2016; Jønsson et al., 2017; Lum et al., 2019; Yang et al., 2020). For example, nuclear IRF3 translocation and subsequent IFN production in HSV-1 infected corneal epithelial cells has been shown to be dependent, at least in part, on p204 expression (Conrady et al., 2012), while TBK-1 phosphorylation and subsequent IFN-β expression by HSV-1 infected HFFs was found to require IFI16 and STING (Orzalli et al., 2012).

Interestingly, Orzalli et al. (2012) used attenuated HSV-1 strains to determine that expression of the immediate early viral protein ICP0 leads to IFI16 degradation and reduced nuclear IRF3 translocation, and this degradation was subsequently shown to be dependent on proteasome activity. In addition, another group has shown that HSV-1 can also lead to IFI16 degradation in an ICP0 independent manner, albeit in the U2OS cell line that lacks a functional STING signaling pathway (Cuchet-Lourenco et al., 2013). Moreover, HSV-1 infection markedly lowers IFI16 protein expression in primary human glia and immortalized cells lines, and this observation may explain why knockdown of this DNA sensor fails to alter viral production or IFN-β release by these cells (Jeffries et al., 2020). Such findings are supported in HFFs where IFI16 knockdown does not affect HSV-1 levels in cells infected with wild type HSV-1 but significantly increases viral titers following infection with an ICP0 null mutant virus (Diner et al., 2016). Similarly, IFI16 has been reported to be dispensable for IFN production in mice following HCMV infection (Gray et al., 2016) and this apparent independence may also stem from the reported ability of HCMV to interfere with IFI16 signaling (Dell'Oste et al., 2014). However, it should be noted that the ability of these viruses to reduce IFI16 protein abundance and/or signaling may show cell type specificity as HSV-1 does not elicit such effects in either HeLa cells or U2OS cells, again perhaps due to a lack of a functional STING signaling pathway in the latter (Orzalli et al., 2016; Deschamps and Kalamvoki, 2017).

Since the available evidence indicates that IFI16 has a role in virally-induced IFN signaling and that this is accomplished through via a STING-dependent pathway, it is possible that this molecule could work in concert with cGAS to stimulate antiviral responses. Evidence for this notion comes from the observation that knockdown of either STING, IFI16, or cGAS, in human fibroblasts leads to reduced HSV-1 infection-induced IFN-β expression (Orzalli et al., 2015). Interestingly, in the same study it was noted that cGAS knockdown reduces constitutive IFI16 protein expression and that this effect was dependent on proteasome activity (Orzalli et al., 2015). This suggests that cGAS may stabilize IFI16 protein levels to promote antiviral activity. However, we found no observable difference in IFI16 protein expression in cGAS deficient microglia created with CRISPR/Cas9 approaches (Jeffries et al., 2020) and so it is possible that, like viral ICP0-mediated effects (Orzalli et al., 2016), cGAS-mediated IFI16 stabilization may show cell type specificity.

Additional support for cooperation between IFI16 and cGAS comes from the work of Jønsson et al. (2017) who demonstrated that IFI16 knockdown reduces cGAMP production by THP-1 cells following foreign DNA challenge. Furthermore, they showed that HEK 293T cells stably expressing IFI16 produce higher amounts of cGAMP following intracellular administration of a cGAS expression plasmid than IFI16 deficient cells (Jønsson et al., 2017). Similarly, another study showed that the co-transfection of increasing amounts of an IFI16 expression plasmid with constant levels of STING and cGAS increased IFN-β activity as assessed by IFN promoter driven luciferase activity, and demonstrated the ability of IFI16 and cGAS to interact directly (Almine et al., 2017). It should be noted that these investigators failed to detect significant changes in cGAMP levels in the absence or presence of IFI16 (Almine et al., 2017). Rather, they determined that IFI16 was required for cGAMP to fully activate STING as assessed by CCL5 expression, STING dimerization, and IRF3 nuclear translocation, in response to cGAMP transfection (Almine et al., 2017).

In contrast to such studies that suggest IFI16 acts in concert with cGAS to promote IFN responses, we demonstrated that cGAS knockdown decreased IFN-β production by a human microglial cell line following DNA transfection but IFI16 knockdown did not (Jeffries et al., 2020). Furthermore, we showed that IFI16 knockdown failed to exacerbate the reduction in IFN-β production by DNA stimulated cGAS deficient microglia, and combined cGAS and IFI16 deficiency failed to significantly alter microglial susceptibility to HSV-1 infection over cGAS deficiency alone (Jeffries et al., 2020). However, an explanation for these results may again stem from the ability of HSV-1 to downregulation IFI16 and cGAS expression and/or inhibit their signaling pathways in human microglia following infection (Jeffries et al., 2020). As such, the development of therapeutics that stabilize the expression of either of these sensor proteins could prove to be an attractive approach to combat the devastating consequences of conditions such as HSV-1 encephalitis.

While cGAS and IFI16 might play redundant roles in STING activation, some evidence suggests that they promote similar responses through different mechanisms. For instance, it was found that IFI16 is not required for cGAS/STING/TBK-1 signaling in HFFs following HSV-1 or HCMV infection, but was for the transcription of IFN-β, ISG54, ISG56, and RANTES (Diner et al., 2016). Interestingly, the same investigators found that IFI16 also reduced the transcription of the HSV-1 genes icp27, icp8, and ul30 (Diner et al., 2016). This suggests that the antiviral functions previously attributed to IFI16 may occur through transcriptional regulation, rather than by direct activation of cGAS-STING signaling. However, our own investigations of the role of IFI16 in HSV-1 transcription in infected human microglial cells showed no discernable effect on icp8 expression (Jeffries et al., 2020). This apparent discrepancy is likely due to differences in the HSV-1 strain employed, as the earlier study used an ICP0 mutant strain that prevents IFI16 degradation, while our studies were performed with the neuroinvasive MacIntyre strain (Diner et al., 2016; Jeffries et al., 2020).

The ability of IFI16 to negatively regulate viral transcription has been reported for other herpesviruses, human papillomavirus (HPV), and hepatitis B virus (HBV) (Gariano et al., 2012; Lo Cigno et al., 2015; Roy et al., 2016, 2019; Pisano et al., 2017; Yang et al., 2020), and the modification of heterochromatin and euchromatin appears to be the primary mechanism by which this is accomplished. For example, U2OS cells or an immortalized human keratinocyte cell line (NIKS) overexpressing IFI16 exhibit elevations in heterochromatin markers, such as H3K9m2, and decreases in euchromatin markers, such as H3K4me2, in early and late HPV promoters as determined by chromatin immunoprecipitation (ChiP) analysis (Lo Cigno et al., 2015). In addition, IFI16 has been demonstrated to directly interact with the histone H3-K9 methyltransferases, SUV39h1 and G9a-like protein (GLP), and knockdown of these proteins in a B cell lymphoma latently infected with KSHV (BCBL1 cells) led to increased viral transcription (Roy et al., 2019). Furthermore, in the same study, IFI16 knockdown reduced the recruitment of both methyltransferases to the KSHV genome (Roy et al., 2019).

In a HBV covalently closed circular DNA (cccDNA) model of infection, overexpression of IFI16 has been shown to increase IFN-β and ISG expression along with decreased euchromatin and increased heterochromatin markers on cccDNA (Yang et al., 2020). Interestingly, knockdown of IFI16 in BCBL1 cells increased the transcription of immediate early, early, and late lytic KSHV genes, indicating reactivation of the lytic cycle, while the reintroduction of IFI16 reduced KSHV genome copy numbers (Roy et al., 2016). This was also found to be true for Akata and MUTU1 cell lines latently infected with Epstein-Barr virus (Pisano et al., 2017). As such, it will be important to determine whether IFI16 similarly contributes to HSV-1 latency in CNS cell types, since reactivation of latent infections is a key event in the onset of HSV-1 encephalitis (Menendez and Carr, 2017). If so, IFI16 could be a promising new therapeutic target, either as an intervention during CNS infection or to prevent reactivation of HSV-1 in at-risk populations.

## ZBP1

Z-DNA binding protein 1 [ZBP1; also known as DNA-dependent activator of interferon regulatory factors (DAI) and DLM-1] was the first identified cytosolic DNA sensor, and was shown to directly bind dsDNA in murine L929 fibroblast-like cells (Takaoka et al., 2007). Importantly, this study demonstrated ZBP1 can interact with TBK1 and IRF3 and contribute to IFN-β mRNA expression following DNA transfection or infection with HSV-1 (Takaoka et al., 2007). However, it seems that this cytosolic DNA sensor may function in a cell type and ligand specific manner, as ZBP1 knockdown in mouse embryonic fibroblasts (MEFs) has little or no effect on exogenous DNA-induced IFN-β expression (Wang et al., 2008). Similarly, ZBP1 knockdown was found to significantly reduce IFN-β expression in L929 cells in response to BDNA transfection but had no effect in a similarly challenged human lung epithelial cell line (Lippmann et al., 2008).

In addition to the expression of antiviral cytokines, ZBP1 has also been shown to mediate the expression of the pro-inflammatory cytokine IL-6 (Takaoka et al., 2007; Kaiser et al., 2008) subsequently demonstrated the activation of a NF-kB-driven luciferase promoter in HEK 293T cells overexpressing ZBP1. This group identified three RIP homotypic interaction motif (RHIM)-like repeats and hypothesized that such NF-κB activation occurs via a RHIM-dependent interaction with receptor interacting protein 1 (RIP1) in a similar manner to that seen with TLR3 (Kaiser et al., 2008). This hypothesis was confirmed by the demonstration that ZBP1 can interact with both RIP1 and receptor interacting protein 3 (RIP3) through its first RHIM domain, and by the ability of RIP1 knockdown or mutations in the RHIM domain in RIP1 or ZBP1 to decrease NF-kB promoter activation. The ability of ZBP1 to interact with RIP1 and RIP3, and to activate NF-kB-mediated gene transcription was subsequently confirmed in a similar HEK 293 cell expression system (Rebsamen et al., 2009). In is interesting to note, however, that co-expression of ZBP1 and RIP3 was also reported to elicit NF-kB activation in these studies, an observation that is in contrast to TLR3 signaling where RIP3 blocks RIP1-mediated NF-kB activation (Kaiser et al., 2008).

In agreement with these studies in non-CNS cell types, we have determined that murine microglia and astrocytes express ZBP1 in an inducible manner, and found that this sensor contributes to pro-inflammatory cytokine production by glia following HSV-1 infection (Furr et al., 2011). Furthermore, these studies also showed that HSV-1 infection induces the production of soluble neurotoxic mediators by astrocytes and microglia in a ZBP1-dependent manner (Furr et al., 2011). Surprisingly, combined knockdown of ZBP1 and retinoic acid inducible gene 1 (RIG-I) leads to greater reductions in TNF-a and IL-6 production by HSV-1 infected glia than either alone, suggesting that these dissimilar sensors can act in synergy (Crill et al., 2015). Together, these studies suggest a role for ZBP1 in inflammation and/or antiviral immunity both in the periphery and the CNS.

While the available evidence supports a role for ZBP1 as a DNA sensor capable of inducing cytokine production, some studies suggest that ZBP1 plays a broader role in antiviral immunity. For example, ZBP1 has been reported to work in concert with RIP3 in murine fibroblasts and epithelial cells to induce necroptosis following infection with a mutant murine cytomegalovirus (MCMV) strain (Upton et al., 2012) that lacks the expression of m45, a viral product that limits ZBP1/RIP3 interactions due the presence of a RHIM domain (Table 1) (Rebsamen et al., 2009; Upton et al., 2012). Interestingly, a similar immune evasion mechanism has been observed for HSV-1 (Table 1), where the viral protein ICP6 also contains a RHIM domain that is capable of inhibiting necroptosis in peripheral human cells (Guo et al., 2015). Necroptotic cell death initiated by simultaneous treatment with TNF-a and the caspase inhibitor zVAD-FMK was blocked following infection with wild type HSV-1, while an ICP6 deficient HSV-1 strain failed to prevent cell death (Guo et al., 2015; Sawai, 2016). This finding is in sharp contrast to studies in mouse cells, where infection with wild type HSV-1 elicits cell death in a RIP3-dependent manner (Wang et al., 2014; Huang et al., 2015). Surprisingly, expression of ICP6 in MEFs was found to be enough to induce RIP3-dependent cell death, while the presence of ICP6 containing mutations in the RHIM domain did not (Wang et al., 2014; Huang et al., 2015), suggesting that ICP6 may be able to directly induce necroptosis in this cell type (Wang et al., 2014). The reason for these apparently contradictory findings was discovered in more recent studies that show ICP6 has species-dependent effects, inducing necroptosis in cells from mice while inhibiting it in human cells, HSV's primary natural host (Huang et al., 2015; Guo et al., 2018).

Importantly, ZBP1 has been found to be a major contributor to necroptosis in both human and mouse fibroblasts following infection with both an ICP6-deficient and an ICP6 RHIM mutant HSV-1 strain (Guo et al., 2018) and our own observations suggest that this cytosolic DNA sensor functions as a mediator of cell death during HSV-1 infection in glia. Our studies indicate that ZBP1 plays a crucial role in triggering necroptosis in murine glia following infection with a strain of HSV-1 that contains mutations in the ICP6 RHIM domain (unpublished observations). The potential importance of this pathway in antiviral immunity within the CNS is underscored by the decreased survival and increased viral burden in the brain of RIP3 deficient mice following HSV-1 infection (Wang et al., 2014; Huang et al., 2015). However, since necroptosis promotes inflammation, it will be important to determine whether this ZBP-mediated response also contributes to CNS pathology during HSV-1 encephalitis, especially in the human host (Dhuriya and Sharma, 2018).

While it is increasingly clear that ZBP1 is a PRR that is capable of initiating cell death pathways, it is less certain what ligands specifically initiate such as response. ZBP1 was initially shown to directly bind BDNA and it has recently been shown to recognize plasmid DNA (Wang et al., 2008; Semenova et al., 2019). However, other studies have shown that ZBP1 is critical for the induction of necroptosis, pyroptosis, and apoptosis, in cells challenged with influenza virus, a segmented negative strand RNA virus (Kuriakose et al., 2016; Thapa et al., 2016), and this role is discussed in depth elsewhere (Dhuriya and Sharma, 2018). By pharmacologically inhibiting various stages of the MCMV life cycle, Sridharan et al. (2017) were able to determine that active transcription was required for ZBP1-mediated cell death, suggesting that RNA serves as the activating ligand in this response. This notion was subsequently supported by two studies describing the ability of ZBP1 to bind endogenous RNA (Maelfait et al., 2017; Jiao et al., 2020).

An ability to sense both RNA and DNA accounts for the protective role of ZBP1 in influenza virus infection and following exposure to other RNA viruses including West Nile virus (WNV) and ZIKV (Daniels et al., 2019; Rothan et al., 2019). Interestingly, however, ZBP1-mediated protection against these neurotropic flaviviruses appears to be independent of cell death in neurons (Daniels et al., 2019; Rothan et al., 2019). Mice genetically deficient in ZBP1 show worse clinical scores, higher viral burdens in the brain, and increased mortality, following WNV infection than wild type mice (Rothan et al., 2019). Similarly, higher viral burdens and mortality have been observed in WNV challenged RIP3 deficient animals, and this effect was independent of cell death pathways (Daniels et al., 2017). Surprisingly, ZBP1 deficient animals demonstrate higher levels of antiviral and inflammatory cytokines/chemokines following WNV infection (Rothan et al., 2019), and this finding is in contrast to similarly infected RIP3 deficient mice, which demonstrate decreased inflammatory cytokine production (Daniels et al., 2017). As such, it is possible these two molecules have independent roles during neuronal infection, especially since peripheral cells undergo cell death in both a ZBP1 and a RIP3-dependent manner (Daniels et al., 2017; Rothan et al., 2019).

A potential mechanism for the antiviral effects of ZBP1 and RIPK3 during neurotropic RNA virus infections comes from the studies of Daniels et al. (2019) that indicate a neuron-specific function for ZBP1. They found that ZBP1-induced antiviral gene expression in neurons following ZIKV infection occurs in a RIP1 and RIP3-dependent manner, and that loss of any of these signaling molecules results in increased viral burden and mortality (Daniels et al., 2019). Surprisingly, RIP3 deficiency in primary microglial cultures did not result in increased ZIKV replication in these studies suggesting that such protection is intrinsic to neurons. Consistent with this notion, upregulation of the antiviral gene *IRG1* was required for protection against both ZIKV and WNV infection in neurons, but this gene was not upregulated in microglia (Daniels et al., 2019), indicating a cell type specific function for ZBP1. Since we have identified a role for this sensor in glia following HSV-1 infection, it will be interesting to see what role, if any, ZBP1 plays in glial responses to neurotropic RNA viruses (Furr et al., 2011; Crill et al., 2015). Regardless, it is apparent that ZBP1 is an important mediator of CNS innate immune responses to both RNA and DNA viruses.

## AIM2

Absent in melanoma 2 (AIM2) is another member of the PHYIN family of interferon inducible proteins that has been found to act as a DNA sensor (Bürckstümmer et al., 2009; Fernandes-Alnemri et al., 2009; Hornung et al., 2009; Adamczak et al., 2014). However, unlike IFI16, recognition of dsDNA by AIM2 has been shown to lead exclusively to inflammasome activation and the induction of pyroptosis, an inflammatory form of cell death (Adamczak et al., 2014). Upon binding to dsDNA, AIM2 associates with the downstream signaling molecule apoptosisassociated speck-like protein containing a CARD domain (ASC), which recruits, and activates caspase-1 (Bürckstümmer et al., 2009; Fernandes-Alnemri et al., 2009; Hornung et al., 2009). Caspase-1 then acts as the effector protein to cleave the immature form of IL-1β and IL-18, leading to the maturation and secretion of these potent inflammatory cytokines (Miao et al., 2011). Additionally, caspase-1 can cleave gasdermin D to initiate pyroptotic cell death, characterized by the formation of pores in the plasma membrane and the release of cellular contents into the extracellular environment (Kayagaki et al., 2015; Shi et al., 2015). The AIM2 inflammasome has been shown to form following infection with either DNA or RNA viruses in peripheral myeloid and lymphoid immune cell-types, such as bone marrow derived dendritic cells (BMDCs), bone marrow derived macrophages (BMDM), monocytes, and fibroblasts (Rathinam et al., 2010; Ekchariyawat et al., 2015; Schattgen et al., 2016; Huang et al., 2017; Zhang et al., 2017), and the role of this and other inflammasomes during viral infection is discussed extensively elsewhere (Chen and Ichinohe, 2015; Lupfer et al., 2015; Man et al., 2016; Shrivastava et al., 2016; Lugrin and Martinon, 2018; Zhu et al., 2019).

While the AIM2 inflammasome is recognized to have an antiviral function in peripheral cell types, relatively little is known about its role in CNS infections despite having been shown to be expressed in neurons and glia (Adamczak et al., 2014; Cox et al., 2015). Furthermore, AIM2 has been shown to function as a DNA sensor in neurons as the cytosolic administration of exogenous DNA induces the association of AIM2 with ASC and leads pyroptosis in these cells (Adamczak et al., 2014). Since neuronal cell death is typically detrimental to the host, it appears likely that the proinflammatory nature of the AIM2 inflammasome can be damaging in the context of viral CNS infections. Circumstantial evidence supporting this notion comes from the effect of deleting ataxia-telangiectasia mutated (ATM), a protein known for its role in activating DNA damage responses, in primary murine microglia (Song et al., 2019). Such a deletion results in cytoplasmic DNA accumulation and cellular activation as demonstrated by the retraction of processes (Song et al., 2019). Moreover, co-culture of microglia and neurons with an ATM inhibitor leads to neuronal cell damage, which is reversed with an IL-1 receptor antagonist consistent with a major role for the inflammasome in this effect (Song et al., 2019). Importantly, this study utilized co-immunoprecipitation approaches to demonstrate that inflammasome activation as a result of ASC association with AIM2 rather than other initiator molecules such as NLR family pyrin domain containing 3 (NLRP3) (Song et al., 2019). A detrimental role for AIM2 in CNS pathologies is further supported by the observation that AIM2 deficient mice show less brain atrophy and cognitive defects following stroke than their wild type counterparts (Kim et al., 2020). Furthermore, caspase-1 inhibition resulted in a similar phenotype in these studies indicating that the improved outcome was due to reduced AIM2 inflammasome activation (Kim et al., 2020).

Despite such evidence, some studies suggest that AIM2 can play a protective role in some infections. For example, the neurotropic RNA viruses, WNV and Chikungunya virus (CHIKV), have been shown to activate the AIM2 inflammasome in peripheral dermal fibroblasts and AIM2 knockdown led to increased CHIKV genome copies in these cells (Ekchariyawat et al., 2015). Furthermore, the higher levels of AIM2, caspase 1, IL-1β, and IL-18, found in brain tissue from still births following ZIKV infection provides circumstantial evidence for a role for this sensor (de Sousa et al., 2018). However, it should be noted that the expression of two other inflammasome activators, NLRP3, and NLRP1, were also elevated in this tissue, and it is not clear whether the upregulation of inflammasome components reflect a protective host response or contribute to disease pathology. Similarly, AIM2 expression is upregulated in neurons following infection with enterovirus A71, the causative agent of hand foot and mouth disease and AIM2 knockdown in a neuronal cell line led to decreased IL-1β cleavage and increased viral copy numbers (Yogarajah et al., 2017). Yet, no mechanism has yet been defined for AIM2mediated sensing of RNA viruses.

With regard to neurotropic DNA viruses, AIM2 was initially demonstrated to be dispensable for inflammasome activation following HSV-1 infection in peritoneal macrophages, but was for necessary for such responses to MCMV challenge (Rathinam et al., 2010). Conversely, another study indicated that IFI16 and NLRP3 were the initiators of inflammasome activation in HFFs following HSV-1 infection (Johnson et al., 2013). This apparent discrepancy may be due to the ability of the HSV-1 product VP22 to block AIM2-mediated inflammasome activation by preventing oligomerization (Table 2) (Maruzuru et al., 2018). Intracranial administration of an HSV-1 strain lacking VP22 leads to decreased viral burdens in wildtype mice but not those lacking AIM2, suggesting that this sensor can limit viral replication in the CNS (Maruzuru et al., 2018). Interestingly, the protective functions of AIM2 in the CNS may extend to bacterial pathogens as AIM2 has been shown to contribute to survival following CNS infection with *Staphylococcus aureus* (Hanamsagar et al., 2014).

It is clear from the available data that out current understanding of the role of AIM2 in the CNS is rudimentary. While some evidence suggests that AIM2 contributes to CNS disease pathology, some indicate protective functions. As such, it may be that AIM2 plays a context-dependent role where this molecule exacerbates sterile inflammation in neurodegenerative diseases when activation tends to be chronic, while acute activation assists in viral or bacterial clearance. Since our understanding of AIM2 in the CNS is based mostly on circumstantial evidence, further study is clearly required to determine the role of this molecule relative to other inflammasome activators, and to determine whether this pathway can be targeted for therapeutic intervention.

## DDX41, Ku70/DNA-PK, and, RNA Polymerase III

Several other putative DNA sensors have been identified in peripheral cell types but their role as PRRs in the CNS remains more controversial. DEAD-Box Helicase 41 (DDX41) was first identified as a cytosolic DNA sensor in a murine dendritic cell-like line with the demonstration that this molecule can directly bind dsDNA and interact with the common DNA sensing and antiviral signaling components STING and TBK1 (Zhang Z. et al., 2011). Importantly, DDX41 knockdown was shown to decrease IFN-α production in these cells in response to dsDNA transfection or infection with either HSV-1 or adenovirus (Zhang Z. et al., 2011). Interestingly, DDX41 has also been shown to directly bind cyclic dinucleotides such as cyclic dimeric guanosine monophosphate (c-di-GMP) and cyclic dimeric adenosine monophosphate (c-diAMP), and knockdown of DDX41 prevents STING association with TBK1 or IRF3 and reduces antiviral signaling in response to these molecules (Parvatiyar et al., 2012). As such, it is possible that DDX41 bolsters cGAS-STING signaling by promoting cGAMP- STING interactions. Evidence for such a suggestion comes from the demonstration that DDX41 knockdown further reduces murine leukemia virus (MLV)-induced IFN expression by cGAS deficient macrophages and dendritic cells (Stavrou et al., 2015). Furthermore, IFN-β expression could be rescued in macrophage-like cells following cGAS knockdown with the administration of exogenous cGAMP prior to MLV infection, but this procedure failed to rescue such responses in cells following DDX41 knockdown (Stavrou et al., 2018). Together, these studies suggest that DDX41 can act in a cooperative manner with cGAS to induce STING activation following viral challenge.

To date, it is not known whether DDX41 is expressed in the mammalian CNS. However, the drosophila DDX41 homolog, Abstrakt, has been shown to be involved in visual and CNS system development (Irion and Leptin, 1999; Schmucker et al., 2000). Furthermore, DDX41 is highly expressed in the zebrafish brain and this gene product performs similar antiviral functions to mammalian DDX41 when expressed in a HEK 293 expression system (Ma et al., 2018). Finally, circumstantial evidence of a role for DDX41 in the human CNS lays in the observation that HSV-1 has evolved an evasion mechanism targeting DDX41 (Table 2), suggesting that this molecule can act as a restriction factor for this neurotropic virus (Duan et al., 2019).

DNA protein kinase (DNA-PK) is a protein complex made up of a DNA protein kinase catalytic subunit (DNA-PKcs), ku70, and ku80, and is best known for its role in DNA double stranded break repair. However, several studies have shown that it can bind to transfected DNA and elicit the expression of IFN-β and other ISGs, independent of kinase activity (Ferguson et al., 2012; Harnor et al., 2017; Burleigh et al., 2020). In addition, the Ku70 subunit has also been identified as a possible DNA sensor in studies where plasmid transfected HEK 293 cells produce the type three IFN, IFN-λ1, that can limit HIV replication (Zhang X. et al., 2011). In this work, Ku70 and Ku80 were both found to bind transfected DNA, but only the loss of Ku70 decreased IFN-λ1 expression in these cells (Zhang X. et al., 2011). This finding was confirmed in splenocytes derived from ku70 deficient mice (Zhang X. et al., 2011), and it was later determined that Ku70mediated IFN-λ1 expression requires the expression of STING (Sui et al., 2017). However, it should be noted that these results are in contrast to a more recent report in which signaling through DNA-PK was found to be independent of the presence of STING (Burleigh et al., 2020). While it is presently unclear whether Ku70 functions alone or in concert with Ku80 and the DNA-PKcs to elicit antiviral activity, Ku70/DNA PK has been demonstrated to induce cytokine responses following infection of hepatocyte carcinoma and monocytic cell lines with HBV and human T-cell leukemia virus type 1, respectively (Li et al., 2016; Wang et al., 2017).

Furthermore, vaccinia virus and adenovirus have both been shown to antagonize Ku70/DNA-PK signaling (Peters et al., 2013; Scutts et al., 2018; Burleigh et al., 2020). Together, these studies suggest that ku70/DNA-PK acts as a viral PRR in addition to its DNA repair functions.

Despite evidence for antiviral functions of ku70/DNA-PK in peripheral cell types such as monocytes and fibroblasts, little exists for such a role in the CNS (Li et al., 2016; Burleigh et al., 2020). Expression of DNA-PK in the CNS and its role in DNA repair has been established from the study of mutations in severe combined immunodeficiency (SCID) mice that result in a truncated kinase domain in DNA-PK (Chechlacz et al., 2001; Vemuri et al., 2001). This has been shown to cause increased neuronal cell death *in vitro* and *in vivo*, presumably as a consequence of accumulated dsDNA breaks (Chechlacz et al., 2001; Vemuri et al., 2001). Since the DNA repair functions of DNA-PK are found in CNS cell types, the DNA sensing abilities of this molecule seen in peripheral cells may also be retained in the brain and this possibility requires further investigation.

Finally, RNA polymerase III was simultaneously identified as a DNA sensor by two groups as they investigated the mechanisms responsible for RIG-I mediated DNA sensing (Ablasser et al., 2009; Chiu et al., 2009). They demonstrated that poly (dA:dT) was reverse transcribed to RNA that then served as a ligand to activate RIG-I and induce IFN-β production in monocytes, fibroblasts, and dendritic cells (Ablasser et al., 2009; Chiu et al., 2009). Our own work subsequently showed that RNA polymerase III is functionally expressed in murine glia and a human microglial cell line (Crill et al., 2015; Johnson et al., 2020), and that its inhibition reduces HSV-1-induced IRF3 activation and TNF-a production in murine microglia cells and astrocytes (Crill et al., 2015). In addition to HSV-1, evidence suggests that RNA polymerase III also has a role in recognizing varicella-zoster virus (VZV) (Carter-Timofte et al., 2018, 2019). A RNA polymerase III mutation was identified in twins suffering from recurrent VZV CNS vasculitis and PBMCs isolated from them showed reduced antiviral and/or inflammatory cytokine responses to poly(dA:dT) and VZV challenge (Carter-Timofte et al., 2018). Furthermore, additional RNA polymerase III mutations were identified in adult VZV encephalitis patients and PBMCs from these patients similarly showed reduced IFN-β and CXCL10 expression in response to poly(dA:dT). Interestingly, while PBMC cytokine response were unchanged following VZV challenge, patients with these RNA polymerase III mutations showed higher viral gene expression (Carter-Timofte et al., 2019). Together, these studies support a role for RNA polymerase III in combating viral CNS infections.

## Concluding Remarks

**Figure 1:**
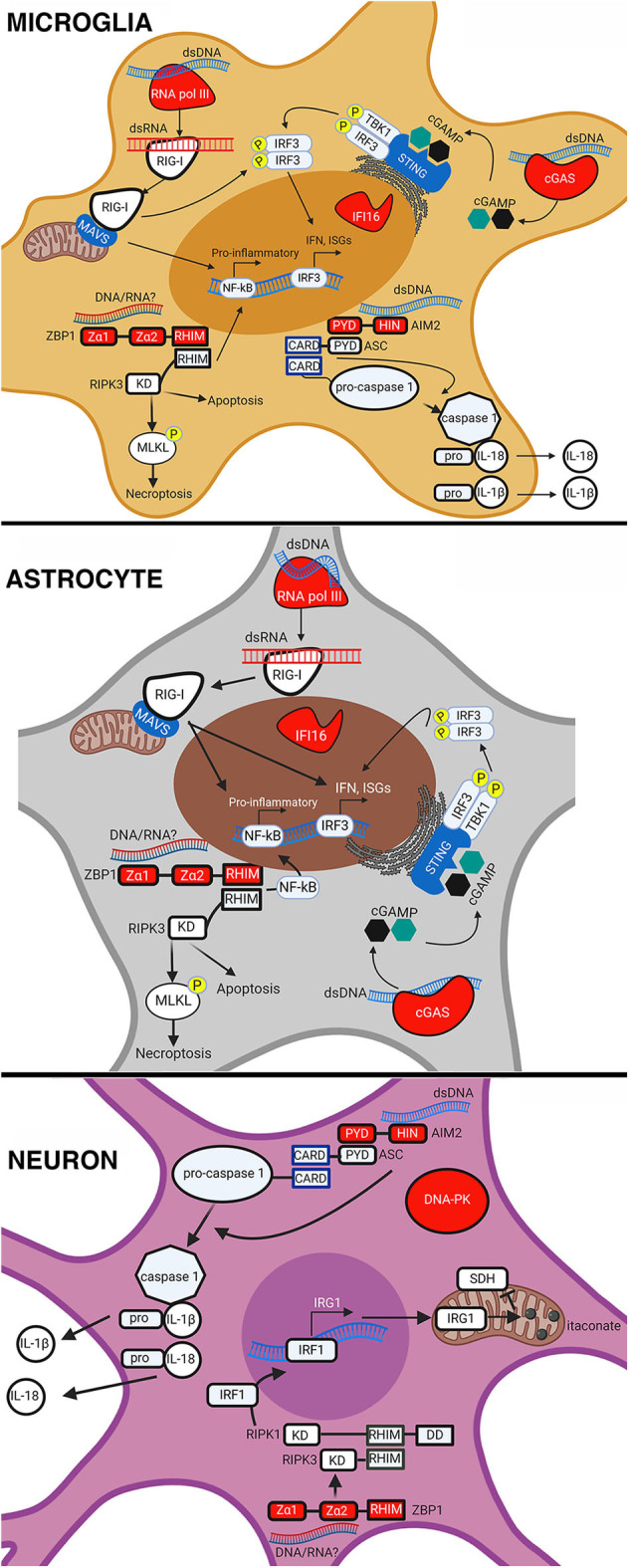
Intracellular DNA sensors in resident CNS cell types. Intracellular nucleic acid sensing by DNA sensors in microglia, astrocytes, and neurons. AIM2 sensing of dsDNA leads to the recruitment of apoptosis-associated speck-like protein containing a CARD (ASC) that then cleaves pro-caspase 1. Active caspase 1 then cleaves the precursor forms of IL18 and IL-1β, causing their maturation and release from the cell. ZBP1 sensing of either dsDNA or RNA causes it to associate with RIPK3, activate the transcription factor NF-κB, and phosphorylate mixed lineage kinase domain-like protein (MLKL) in microglia and astrocytes. This results in pro-inflammatory cytokine expression and execution of necroptosis. In neurons, ZBP1 sensing results in the activation of IRF1, expression of IRG1, production of itaconate, and a reduction in succinate dehydrogenase (SDH) activity. Sensing of dsDNA by cGAS leads to the production of cGAMP, which binds to and activates STING causing the phosphorylation and translocation of interferon regulatory factor 3 (IRF3) to the nucleus. This results in the expression of IFN and ISGs in microglia and astrocytes. RNA pol III senses dsDNA and converts it into dsRNA that can then be sensed by RIG-I in microglia and astrocytes. RIG-I sensing of dsRNA causes it to associate with mitochondrial antiviral-signaling protein (MAVS) leading to activation and translocation of IRF3 and NF-κB, resulting in the expression of IFN, ISGs, and pro-inflammatory cytokines. This figure was created with BioRender.com.

It is now appreciated that resident CNS cells are important contributors to innate immunity and, due to their location, are likely the first responders to viral CNS infections. Resident CNS cells, especially astrocytes and microglia, are known to express an array of PRRs including TLRs, RLRs, NLRs, and now intracellular DNA sensors. In addition to their expression, multiple studies have demonstrated the functional nature of these sensors in various CNS cell types (as summarized in Figure 1 and Table 3). For example, we have shown that cGAS is required, at least in part, for microglial IFN responses to foreign DNA (Jeffries et al., 2020). However, our understanding of the role of DNA sensors in viral infections is limited and, in some cases, contradictory. It is currently unclear whether DNA sensors are beneficial or detrimental to the host during CNS infections, and it appears likely that outcomes following activation are pathogen, host cell-type, and even species, specific. This is exemplified by the finding that the HSV-1 product ICP6 blocks ZBP1-mediated responses in human cells but activates ZBP1 in murine cells (Guo et al., 2018). Because of this, future research on the role and therapeutic potential of DNA sensors must be cognizant of such variables.

**Table 3:** Expression and antiviral activity of intracellular DNA sensors in CNS cell types.

**Sensor**	**CNS cell type**	**Antiviral activity**	**References**
ZBP1	Primary mouse whole brain tissue, cortical neurons, microglia, and astrocytes	Neuronal immunometabolism regulation, antiviral, and proinflammatory cytokine production	Furr et al., 2011; Daniels et al., 2019; Rothan et al., 2019
cGAS	Primary murine neurons, astrocytes, and microglia Primary human astrocytes and microglia Human cell lines U87-MG and hμglia	IFN and ISG expression	Cox et al., 2015; Reinert et al., 2016; Jeffries and Marriott, 2017
IFI16	Primary mouse epithelial cells Primary human astrocytes Human cell lines, corneal epithelial, astrocytes, microglia	Inflammasome activation	Conrady et al., 2012; Coulon et al., 2019; Jeffries et al., 2020
AIM2	Primary mouse astrocytes and microglia Human cell line, SK-N-SH	Inflammasome activation	Cox et al., 2015; Yogarajah et al., 2017; Song et al., 2019
DDX41	Zebrafish whole brain	IFN expression	Ma et al., 2018
DNA PK	Primary mouse cerebral cortex and neurons	Unknown	Chechlacz et al., 2001; Vemuri et al., 2001
RNA pol III	Primary mouse astrocytes and microglia Mouse cell line, EOC13.31 Human cell line, hμglia	IFN expression and NF-κB activation via RIG-I	Crill et al., 2015; Johnson et al., 2020

Lastly, it is important to note that DNA sensors may contribute to other CNS pathologies, such as neurodegenerative diseases, which may be initiated or exacerbated by viral infection. For instance, three prime repair exonuclease 1 (TREX1) deficiency can cause accumulation of mislocalized DNA and lead to Aicardi Goutieres syndrome. This condition is characterized by permanent and often severe neurological damage due to IFN overproduction, and the loss of cGAS has been shown to rescue TREX1 deficient mice from disease pathology (Gray et al., 2016). Similarly, neurodegenerative diseases are associated with chronic overproduction of proinflammatory mediators and neuroinflammation in diseases such as Alzheimer's disease could result from the chronic activation of DNA sensors by released DAMPS and/or viral infection. Regardless, it is clear that our understanding of these DNA sensors in the CNS remains rudimentary and further research is needed to define the cell type, species, and pathogen specificity of each. In doing so, it might be possible to target these molecules judiciously to limit damaging inflammation while allowing beneficial host responses to improve patient outcomes.

## Author Contributions

AJ and IM co-wrote this literature review article. Both authors contributed to the article and approved the submitted version.

## Conflict of Interest

The authors declare that the research was conducted in the absence of any commercial or financial relationships that could be construed as a potential conflict of interest.

## References

[B1] Aarreberg L. D. Esser-Nobis K. Driscoll C. Shuvarikov A. Roby J. A. Gale M. (2019). Interleukin-1β Induces mtDNA release to activate innate immune signaling via cGAS-STING. Mol. Cell 74, 801–815.e6. 10.1016/j.molcel.2019.02.038 30952515 PMC6596306

[B2] Abe T. Barber G. N. (2014). Cytosolic-DNA-Mediated, STING-Dependent Proinflammatory gene induction necessitates canonical nf- b activation through TBK1. J. Virol. 88, 5328–5341. 10.1128/JVI.00037-14 24600004 PMC4019140

[B3] Ablasser A. Bauernfeind F. Hartmann G. Latz E. Fitzgerald K. A. Hornung V. (2009). RIG-I-dependent sensing of poly(dA:dT) through the induction of an RNA polymerase III transcribed RNA intermediate. Nat. Immunol. 10, 1065–1072. 10.1038/ni.1779 19609254 PMC3878616

[B4] Ablasser A. Chen Z. J. (2019). CGAS in action: Expanding roles in immunity and inflammation. Science 80:363. 10.1126/science.aat8657 30846571

[B5] Adamczak S. E. De Rivero Vaccari J. P. Dale G. Brand F. J. Nonner D. Bullock M.. (2014). Pyroptotic neuronal cell death mediated by the AIM2 inflammasome. *J*. Cereb. Blood Flow Metab. 34, 621–629. 10.1038/jcbfm.2013.236 24398937 PMC3982080

[B6] Aguirre S. Luthra P. Sanchez-Aparicio M. T. Maestre A. M. Patel J. Lamothe F.. (2017). Dengue virus NS2B protein targets cGAS for degradation and prevents mitochondrial DNA sensing during infection. Nat. Microbiol. 2, 1–11. 10.1038/nmicrobiol.2017.37 28346446 PMC7457382

[B7] Aguirre S. Maestre A. M. Pagni S. Patel J. R. Savage T. Gutman D.. (2012). DENV inhibits type I IFN production in infected cells by cleaving human STING. PLoS Pathog. 8:e1002934. 10.1371/journal.ppat.1002934 23055924 PMC3464218

[B8] Almine J. F. O'Hare C. A. J. Dunphy G. Haga I. R. Naik R. J. Atrih A.. (2017). IFI16 and cGAS cooperate in the activation of STING during DNA sensing in human keratinocytes. Nat. Commun. 8:14392. 10.1038/ncomms14392 28194029 PMC5316833

[B9] Aloisi F. (2000). The role of microglia and astrocytes in CNS immune surveillance and immunopathology. Adv Exp Med Biol, 123–133. 10.1007/978-1-4615-4685-6_10 10635024

[B10] Aloisi F. (2001). Immune function of microglia. Glia 36, 165–179. 10.1002/glia.1106 11596125

[B11] Ansari M. A. Dutta S. Veettil M. V. Dutta D. Iqbal J. Kumar B.. (2015). Herpesvirus genome recognition induced acetylation of nuclear IFI16 Is essential for its cytoplasmic translocation, inflammasome and IFN-β responses. PLoS Pathog. 11:e10050. 10.1371/journal.ppat.1005019 26134128 PMC4489722

[B12] Ansari M. A. Singh V. V. Dutta S. Veettil M. V. Dutta D. Chikoti L. (2013). Constitutive interferon-inducible protein 16-Inflammasome activation during epstein-barr virus latency i, ii, and iii in b and epithelial cells. J. Virol. 87, 8606–8623. 10.1128/JVI.00805-13 23720728 PMC3719826

[B13] Biolatti M. Dell'Oste V. Pautasso S. Gugliesi F. von Einem J. Krapp C.. (2017). Human cytomegalovirus tegument protein pp65 (pUL83) dampens type I interferon production by inactivating the DNA sensor cGAS without affecting STING. J. Virol. 92, e01774–17. 10.1128/JVI.01774-17 29263269 PMC5827387

[B14] Bode C. Fox M. Tewary P. Steinhagen A. Ellerkmann R. K. Klinman D.. (2016). Human plasmacytoid dentritic cells elicit a Type I Interferon response by sensing DNA via the cGAS-STING signaling pathway. Eur. J. Immunol. 46, 1615–1621. 10.1002/eji.201546113 27125983 PMC6389263

[B15] Botto S. Abraham J. Mizuno N. Pryke K. Gall B. Landais I.. (2019). Human cytomegalovirus immediate early 86-kda protein blocks transcription and induces degradation of the immature interleukin-1β protein during virion-mediated activation of the AIM2 inflammasome. MBio 10, e02510–18. 10.1128/mBio.02510-18 30755509 PMC6372796

[B16] Bowman C. C. Rasley A. Tranguch S. L. Marriott I. (2003). Cultured astrocytes express toll-like receptors for bacterial products. Glia 43, 281–291. 10.1002/glia.10256 12898707

[B17] Bradshaw M. J. Venkatesan A. (2016). Herpes simplex virus-1 encephalitis in adults: pathophysiology, diagnosis, and management. Neurotherapeutics 13, 493–508. 10.1007/s13311-016-0433-7 27106239 PMC4965403

[B18] Bsibsi M. Ravid R. Gveric D. van Noort J. M. (2002). Broad expression of toll-like receptors in the human central nervous system. J. Neuropathol. Exp. Neurol. 61, 1013–21. 10.1093/jnen/61.11.1013 12430718

[B19] Bsibsi M. Persoon-Deen C. Verwer R. W. H. Meeuwsen S. Ravid R. Van Noort J. M. (2006). Toll-like receptor 3 on adult human astrocytes triggers production of neuroprotective mediators. Glia 53, 688–695. 10.1002/glia.20328 16482523

[B20] Bürckstümmer T. Baumann C. Blüml S. Dixit E. Dürnberger G. Jahn H.. (2009). An orthogonal proteomic-genomic screen identifies AIM2 as a cytoplasmic DNA sensor for the inflammasome. Nat. Immunol. 10, 266–272. 10.1038/ni.1702 19158679

[B21] Burleigh K. Maltbaek J. H. Cambier S. Green R. Gale M. James R. C.. (2020). Human DNA-PK activates a STING-independent DNA sensing pathway. Sci. Immunol. 5:aba4219. 10.1126/sciimmunol.aba4219 31980485 PMC7081723

[B22] Cai X. Chiu Y. H. Chen Z. J. (2014). The cGAS-cGAMP-STING pathway of cytosolic DNA sensing and signaling. Mol. Cell 54, 289–296. 10.1016/j.molcel.2014.03.040 24766893

[B23] Carter-Timofte M. E. Hansen A. F. Christiansen M. Paludan S. R. Mogensen T. H. (2019). Mutations in RNA Polymerase III genes and defective DNA sensing in adults with varicella-zoster virus CNS infection. Genes Immun. 20, 214–223. 10.1038/s41435-018-0027-y 29728610

[B24] Carter-Timofte M. E. Hansen A. F. Mardahl M. Fribourg S. Rapaport F. Zhang S. Y.. (2018). Varicella-zoster virus CNS vasculitis and RNA polymerase III gene mutation in identical twins. Neurol. Neuroimmunol. NeuroInflammation 5:e500. 10.1101/244848 30211253 PMC6131052

[B25] Carty M. Reinert L. Paludan S. R. Bowie A. G. (2014). Innate antiviral signaling in the central nervous system. Trends Immunol. 35, 79–87. 10.1016/j.it.2013.10.012 24316012

[B26] Cerón S. North B. J. Taylor S. A. Leib D. A. (2019). The STING agonist 5,6-dimethylxanthenone-4-acetic acid (DMXAA) stimulates an antiviral state and protects mice against herpes simplex virus-induced neurological disease. Virology 529, 23–28. 10.1016/j.virol.2019.01.006 30648635 PMC6382592

[B27] Chan Y. K. Gack M. U. (2016). Viral evasion of intracellular DNA and RNA sensing. Nat. Rev. Microbiol. 14, 360–373. 10.1038/nrmicro.2016.45 27174148 PMC5072394

[B28] Chaudhuri A. Kennedy P. G. E. (2002). Diagnosis and treatment of viral encephalitis. Postgrad. Med. J. 78, 575–583. 10.1136/pmj.78.924.575 12415078 PMC1742520

[B29] Chauhan V. S. Sterka D. G. Furr S. R. Young A. B. Marriott I. (2009). NOD2 plays an important role in the inflammatory responses of microglia and astrocytes to bacterial CNS pathogens. Glia 57, 414–423. 10.1002/glia.20770 18803303 PMC2628967

[B30] Chechlacz M. Vemuri M. C. Naegele J. R. (2001). Role of DNA-dependent protein kinase in neuronal survival. J. Neurochem. 78, 141–154. 10.1046/j.1471-4159.2001.00380.x 11432981

[B31] Chen H. He G. Chen Y. Zhang X. Wu S. (2018). Differential activation of NLRP3, AIM2, and IFI16 inflammasomes in humans with acute and chronic hepatitis B. Viral Immunol. 31, 639–645. 10.1089/vim.2018.0058 30222506

[B32] Chen I. Y. Ichinohe T. (2015). Response of host inflammasomes to viral infection. Trends Microbiol. 23, 55–63. 10.1016/j.tim.2014.09.007 25456015

[B33] Chen Q. Sun L. Chen Z. J. (2016). Regulation and function of the cGAS-STING pathway of cytosolic DNA sensing. Nat. Immunol. 17, 1142–1149. 10.1038/ni.3558 27648547

[B34] Cheng W. Y. He X. B. Jia H. J. Chen G. H. Jin Q. W. Long Z. L.. (2018). The cGassting signaling pathway is required for the innate immune response against ectromelia virus. Front. Immunol. 9:1297. 10.3389/fimmu.2018.01297 29963044 PMC6010520

[B35] Chiu Y. H. MacMillan J. B. Chen Z. J. (2009). RNA Polymerase III detects cytosolic DNA and induces type i interferons through the rig-i pathway. Cell 138, 576–591. 10.1016/j.cell.2009.06.015 19631370 PMC2747301

[B36] Choi H. J. Park A. Kang S. Lee E. Lee T. A. Ra E. A.. (2018). Human cytomegalovirus-encoded US9 targets MAVS and STING signaling to evade type i interferon immune responses. Nat. Commun. 9:125. 10.1038/s41467-017-02624-8 29317664 PMC5760629

[B37] Christensen M. H. Jensen S. B. Miettinen J. J. Luecke S. Prabakaran T. Reinert L. S.. (2016). HSV−1 ICP 27 targets the TBK 1-activated STING signalsome to inhibit virus-induced type I IFN expression. EMBO J. 35, 1385–1399. 10.15252/embj.201593458 27234299 PMC4931188

[B38] Conrady C. D. Zheng M. Fitzgerald K. A. Liu C. Carr D. J. J. (2012). Resistance to HSV-1 infection in the epithelium resides with the novel innate sensor, IFI-16. Mucosal Immunol. 5, 173–183. 10.1038/mi.2011.63 22236996 PMC3288395

[B39] Corrales L. Woo S.-R. Williams J. B. McWhirter S. M. Dubensky T. W. Gajewski T. F. (2016). Antagonism of the STING pathway via activation of the AIM2 inflammasome by intracellular DNA. J. Immunol. 196, 3191–3198. 10.4049/jimmunol.1502538 26927800 PMC4800192

[B40] Coulon P. G. Dhanushkodi N. Prakash S. Srivastava R. Roy S. Alomari N. I.. (2019). NLRP3, NLRP12, and IFI16 inflammasomes induction and caspase-1 activation triggered by virulent HSV-1 strains are associated with severe corneal inflammatory herpetic disease. Front. Immunol. 10:1631. 10.3389/fimmu.2019.01631 31367214 PMC6644090

[B41] Cox D. J. Field R. H. Williams D. G. Baran M. Bowie A. G. Cunningham C.. (2015). DNA sensors are expressed in astrocytes and microglia *in vitro* and are upregulated during gliosis in neurodegenerative disease. Glia 63, 812–825. 10.1002/glia.22786 25627810 PMC4657478

[B42] Cridland J. A. Curley E. Z. Wykes M. N. Schroder K. Sweet M. J. Roberts T. L.. (2012). The mammalian PYHIN gene family: Phylogeny, evolution and expression. BMC Evol. Biol. 12:140. 10.1186/1471-2148-12-140 22871040 PMC3458909

[B43] Crill E. K. Furr-Rogers S. R. Marriott I. (2015). RIG-I is required for VSV-induced cytokine production by murine glia and acts in combination with DAI to initiate responses to HSV-1. Glia 63, 2168–2180. 10.1002/glia.22883 26146945 PMC4600648

[B44] Cuchet-Lourenco D. Anderson G. Sloan E. Orr A. Everett R. D. (2013). The Viral Ubiquitin ligase icp0 is neither sufficient nor necessary for degradation of the cellular dna sensor ifi16 during herpes simplex virus 1 infection. J. Virol. 87, 13422–13432. 10.1128/JVI.02474-13 24089555 PMC3838218

[B45] Dai P. Wang W. Cao H. Avogadri F. Dai L. Drexler I.. (2014). Modified Vaccinia Virus ankara triggers type i ifn production in murine conventional dendritic cells via a cGAS/STING-mediated cytosolic DNA-sensing pathway. PLoS Pathog. 10:e1003989. 10.1371/journal.ppat.1003989 24743339 PMC3990710

[B46] Daniels B. P. Kofman S. B. Smith J. R. Norris G. T. Snyder A. G. Kolb J. P.. (2019). The nucleotide sensor zbp1 and kinase RIPK3 induce the enzyme irg1 to promote an antiviral metabolic state in neurons. Immunity 50, 64–76.e4. 10.1016/j.immuni.2018.11.017 30635240 PMC6342485

[B47] Daniels B. P. Snyder A. G. Olsen T. M. Orozco S. Oguin T. H. Tait S. W. G.. (2017). RIPK3 restricts viral pathogenesis via cell Death-Independent Neuroinflammation. Cell 169, 301–313.e11. 10.1016/j.cell.2017.03.011 28366204 PMC5405738

[B48] de Sousa J. R. da Silva Azevedo R. D. S. Martins Filho A. J. de Araujo M. T. F. Cruz E. D. R. M. Vasconcelos B. C. B.. (2018). *In situ* inflammasome activation results in severe damage to the central nervous system in fatal Zika virus microcephaly cases. Cytokine 111, 255–264. 10.1016/j.cyto.2018.08.008 30199767

[B49] Dell'Oste V. Gatti D. Giorgio A. G. Gariglio M. Landolfo S. De Andrea M. (2015). The interferon-inducible DNA-sensor protein IFI16: A key player in the antiviral response. New Microbiol. 38, 5–20. 25742143

[B50] Dell'Oste V. Gatti D. Gugliesi F. De Andrea M. Bawadekar M. Lo Cigno I.. (2014). Innate nuclear sensor ifi16 translocates into the cytoplasm during the early stage of *in vitro* human cytomegalovirus infection and is entrapped in the egressing virions during the late stage. J. Virol. 88, 6970–6982. 10.1128/JVI.00384-14 24696486 PMC4054358

[B51] Deschamps T. Kalamvoki M. (2017). Evasion of the STING DNA-sensing pathway by VP11/12 of herpes simplex virus 1. J. Virol. 91:e00535–17. 10.1128/JVI.00535-17 28592536 PMC5533902

[B52] Dhanwani R. Takahashi M. Sharma S. (2018). Cytosolic sensing of immuno-stimulatory DNA, the enemy within. Curr. Opin. Immunol. 50, 82–87. 10.1016/j.coi.2017.11.004 29247853 PMC5916810

[B53] Dhuriya Y. K. Sharma D. (2018). Necroptosis: a regulated inflammatory mode of cell death. J. Neuroinflammation 15:199. 10.1186/s12974-018-1235-0 29980212 PMC6035417

[B54] Diner B. A. Lum K. K. Javitt A. Cristea I. M. (2015). Interactions of the antiviral factor interferon gamma-inducible protein 16 (IFI16) mediate immune signaling and herpes simplex virus-1 immunosuppression. Mol. Cell. Proteomics 14, 2341–2356. 10.1074/mcp.M114.047068 25693804 PMC4563720

[B55] Diner B. A. Lum K. K. Toettcher J. E. Cristea I. M. (2016). Viral DNA sensors IFI16 and cyclic GMP-AMP synthase possess distinct functions in regulating viral gene expression, immune defenses, and apoptotic responses during herpesvirus infection. MBio 7, e01553–16. 10.1128/mBio.01553-16 27935834 PMC5111403

[B56] Ding Q. Gaska J. M. Douam F. Wei L. Kim D. Balev M.. (2018). Species-specific disruption of STING-dependent antiviral cellular defenses by the Zika virus NS2B3 protease. Proc. Natl. Acad. Sci. U.S.A. 115, E6310–E6318. 10.1073/pnas.1803406115 29915078 PMC6142274

[B57] Duan Y. Zeng J. Fan S. Liao Y. Feng M. Wang L.. (2019). Herpes simplex virus type 1-encoded miR-H2-3p manipulates cytosolic DNA-stimulated antiviral innate immune response by targeting DDX41. Viruses 11:756. 10.3390/v11080756 31443275 PMC6723821

[B58] Dutta D. Dutta S. Veettil M. V. Roy A. Ansari M. A. Iqbal J.. (2015). BRCA1 Regulates IFI16 mediated nuclear innate sensing of herpes viral dna and subsequent induction of the innate inflammasome and interferon-β responses. PLOS Pathog. 11:e1005030. 10.1371/journal.ppat.1005030 26121674 PMC4487893

[B59] Ekchariyawat P. Hamel R. Bernard E. Wichit S. Surasombatpattana P. Talignani L.. (2015). Inflammasome signaling pathways exert antiviral effect against chikungunya virus in human dermal fibroblasts. Infect. Genet. Evol. 32, 401–408. 10.1016/j.meegid.2015.03.025 25847693

[B60] Fang R. Wang C. Jiang Q. Lv M. Gao P. Yu X.. (2017). NEMO–IKKβ are essential for IRF3 and NF-κB activation in the cGAS–STING pathway. J. Immunol. 199, 3222–3233. 10.4049/jimmunol.1700699 28939760

[B61] Ferguson B. J. Mansur D. S. Peters N. E. Ren H. Smith G. L. (2012). DNA-PK is a DNA sensor for IRF-3-dependent innate immunity. Elife 2012:e00047. 10.7554/eLife.00047.012 23251783 PMC3524801

[B62] Fernandes-Alnemri T. Yu J. W. Datta P. Wu J. Alnemri E. S. (2009). AIM2 activates the inflammasome and cell death in response to cytoplasmic DNA. Nature 458, 509–513. 10.1038/nature07710 19158676 PMC2862225

[B63] Fu Y. Z. Su S. Gao Y. Q. Wang P. P. Huang Z. F. Hu M. M.. (2017). Human cytomegalovirus tegument protein UL82 inhibits STING-mediated signaling to evade antiviral immunity. Cell Host Microbe 21, 231–243. 10.1016/j.chom.2017.01.001 28132838

[B64] Furr S. R. Chauhan V. S. Moerdyk-Schauwecker M. J. Marriott I. (2011). A role for DNA-dependent activator of interferon regulatory factor in the recognition of herpes simplex virus type 1 by glial cells. J. Neuroinflammation 8:99. 10.1186/1742-2094-8-99 21838860 PMC3168419

[B65] Furr S. R. Chauhan V. S. Sterka D. Grdzelishvili V. Marriott I. (2008). Characterization of retinoic acid–inducible gene-I expression in primary murine glia following exposure to vesicular stomatitis virus. J. Neurovirol. 14, 503–513. 10.1080/13550280802337217 18991139 PMC3833003

[B66] Furr S. R. Marriott I. (2012). Viral CNS infection: role of glial pattern recognition receptors in neuroinflammation. Front. Microbiol. 3:201. 10.3389/fmicb.2012.00201 22723794 PMC3379540

[B67] Gao D. Wu J. Wu Y. T. Du F. Aroh C. Yan N.. (2013). Cycilc GMP-AMP Synthase is and innate immune sensor of HIV and other retroviruses. Science 341, 903–906. 10.1126/science.1240933 23929945 PMC3860819

[B68] Gao P. Ascano M. Wu Y. Barchet W. Gaffney B. L. Zillinger T.. (2013). Cyclic [G(2′,5′)pA(3′,5′)p]is the metazoan second messenger produced by DNA-activated cyclic GMP-AMP synthase. Cell 153, 1094–1107. 10.1016/j.cell.2013.04.046 23647843 PMC4382009

[B69] Gariano G. R. Dell'Oste V. Bronzini M. Gatti D. Luganini A. de Andrea M.. (2012). The intracellular DNA sensor IFI16 gene acts as restriction factor for human Cytomegalovirus replication. PLoS Pathog. 8:e1002498. 10.1371/journal.ppat.1002498 22291595 PMC3266931

[B70] George B. P. Schneider E. B. Venkatesan A. (2014). Encephalitis hospitalization rates and inpatient mortality in the United States, 2000-2010. PLoS ONE 9:e0104169. 10.1371/journal.pone.0104169 25192177 PMC4156306

[B71] Gray E. E. Winship D. Snyder J. M. Child S. J. Geballe A. P. Stetson D. B. (2016). The AIM2-like receptors are dispensable for the interferon response to intracellular DNA. Immunity 45, 255–266. 10.1016/j.immuni.2016.06.015 27496731 PMC4988931

[B72] Guo H. Gilley R. P. Fisher A. Lane R. Landsteiner V. J. Ragan K. B.. (2018). Species-independent contribution of ZBP1/DAI/DLM-1-triggered necroptosis in host defense against HSV1. Cell Death Dis. 9:816. 10.1038/s41419-018-0868-3 30050136 PMC6062522

[B73] Guo H. Omoto S. Harris P. A. Finger J. N. Bertin J. Gough P. J.. (2015). Herpes simplex virus suppresses necroptosis in human cells. Cell Host Microbe 17, 243–251. 10.1016/j.chom.2015.01.003 25674983 PMC4382104

[B74] Hanamsagar R. Aldrich A. Kielian T. (2014). Critical role for the AIM2 inflammasome during acute CNS bacterial infection. J. Neurochem. 129, 704–711. 10.1111/jnc.12669 24484406 PMC3999210

[B75] Harnor S. J. Brennan A. Cano C. (2017). Targeting DNA-dependent protein kinase for cancer therapy. ChemMedChem 12, 895–900. 10.1002/cmdc.201700143 28423228

[B76] Herzner A. M. Hagmann C. A. Goldeck M. Wolter S. Kübler K. Wittmann S.. (2015). Sequence-specific activation of the DNA sensor cGAS by Y-form DNA structures as found in primary HIV-1 cDNA. Nat. Immunol. 16, 1025–1033. 10.1038/ni.3267 26343537 PMC4669199

[B77] Hornung V. Ablasser A. Charrel-Dennis M. Bauernfeind F. Horvath G. Caffrey D. R.. (2009). AIM2 recognizes cytosolic dsDNA and forms a caspase-1-activating inflammasome with ASC. Nature 458, 514–518. 10.1038/nature07725 19158675 PMC2726264

[B78] Huang J. You H. Su C. Li Y. Chen S. Zheng C. (2018). Herpes simplex virus 1 tegument protein VP22 abrogates cGAS/STING-mediated antiviral innate immunity. J. Virol. 92, e00841–18. 10.1128/JVI.00841-18 29793952 PMC6052299

[B79] Huang Y. Liu L. Ma D. Liao Y. Lu Y. Huang H.. (2017). Human cytomegalovirus triggers the assembly of AIM2 inflammasome in THP-1-derived macrophages. J. Med. Virol. 89, 2188–2195. 10.1002/jmv.24846 28480966

[B80] Huang Z. Wu S. Q. Liang Y. Zhou X. Chen W. Li L.. (2015). RIP1/RIP3 binding to HSV-1 ICP6 initiates necroptosis to restrict virus propagation in mice. Cell Host Microbe. 17, 229–242. 10.1016/j.chom.2015.01.002 25674982

[B81] Huang Z. F. Zou H. M. Liao B. W. Zhang H. Y. Yang Y. Fu Y. Z.. (2018). Human cytomegalovirus protein UL31 inhibits DNA sensing of cGAS to mediate immune evasion. Cell Host Microbe 24, 69–80.e4. 10.1016/j.chom.2018.05.007 29937271

[B82] Ingram J. P. Thapa R. J. Fisher A. Tummers B. Zhang T. Yin C.. (2019). ZBP1/DAI drives RIPK3-mediated cell death induced by IFNs in the absence of RIPK1. J. Immunol. 203, 1348–1355. 10.4049/jimmunol.1900216 31358656 PMC6702065

[B83] Iqbal J. Ansari M. A. Kumar B. Dutta D. Roy A. Chikoti L.. (2016). Histone H2BIFI16 recognition of nuclear herpesviral genome induces cytoplasmic interferon-β responses. PLOS Pathog. 12:e1005967. 10.1371/journal.ppat.1005967 27764250 PMC5072618

[B84] Irion U. Leptin M. (1999). Developmental and cell biological functions of the drosophila DEAD-box protein abstrakt. Curr. Biol. 9, 1373–1381. 10.1016/S0960-9822(00)80082-2 10607561

[B85] Ishikawa H. Ma Z. Barber G. N. (2009). STING regulates intracellular DNA-mediated, type i interferon-dependent innate immunity. Nature 461, 788–792. 10.1038/nature08476 19776740 PMC4664154

[B86] Jakobsen M. R. Bak R. O. Andersen A. Berg R. K. Jensen S. B. Jin T.. (2013). IFI16 senses DNA forms of the lentiviral replication cycle and controls HIV-1 replication. Proc. Natl. Acad. Sci. U.S.A. 110:E4571–80. 10.1073/pnas.1311669110 24154727 PMC3845190

[B87] Jeffries A. M. Marriott I. (2017). Human microglia and astrocytes express cGAS-STING viral sensing components. Neurosci. Lett. 658, 53–56. 10.1016/j.neulet.2017.08.039 28830822 PMC5645252

[B88] Jeffries A. M. Nitika T. A. W. Marriott I. (2020). The intracellular DNA sensors cGAS and IFI16 do not mediate effective antiviral immune responses to HSV-1 in human microglial cells. J. Neurovirol. 26, 544–555. 10.1007/s13365-020-00852-1 32488842 PMC7442602

[B89] Jiao H. Wachsmuth L. Kumari S. Schwarzer R. Lin J. Eren R. O. (2020). Z-nucleicacid sensing triggers ZBP1-dependent necroptosis and inflammation. Nature 580, 391–395. 10.1038/s41586-020-2129-8 32296175 PMC7279955

[B90] Johnson K. E. Chikoti L. Chandran B. (2013). Herpes simplex virus 1 infection induces activation and subsequent inhibition of the IFI16 and NLRP3 Inflammasomes. J. Virol. 87, 5005–5018. 10.1128/JVI.00082-13 23427152 PMC3624293

[B91] Johnson M. B. Halman J. R. Burmeister A. R. Currin S. Khisamutdinov E. F. Afonin K. A.. (2020). Retinoic acid inducible gene-I mediated detection of bacterial nucleic acids in human microglial cells. J. Neuroinflammation 17:139. 10.1186/s12974-020-01817-1 32357908 PMC7195775

[B92] Jønsson K. L. Laustsen A. Krapp C. Skipper K. A. Thavachelvam K. Hotter D.. (2017). IFI16 is required for DNA sensing in human macrophages by promoting production and function of cGAMP. *Nat*. Commun. 8:14391. 10.1038/ncomms14391 28186168 PMC5309897

[B93] Kaiser W. J. Upton J. W. Mocarski E. S. (2008). Receptor-interacting protein homotypic interaction motif-dependent control of NF-κB activation via the DNA-dependent activator of IFN regulatory factors. J. Immunol. 181, 6427–6434. 10.4049/jimmunol.181.9.6427 18941233 PMC3104927

[B94] Kayagaki N. Stowe I. B. Lee B. L. O'Rourke K. Anderson K. Warming S.. (2015). Caspase-11 cleaves gasdermin D for non-canonical inflammasome signalling. Nature 526, 666–671. 10.1038/nature15541 26375259

[B95] Kim H. Seo J. S. Lee S. Y. Ha K. T. Choi B. T. Shin Y.. (2020). AIM2 inflammasome contributes to brain injury and chronic post-stroke cognitive impairment in mice. Brain. Behav. Immun. 87, 765–776. 10.1016/j.bbi.2020.03.011 32201254

[B96] Kim J. E. Kim Y. E. Stinski M. F. Ahn J. H. Song Y. J. (2017). Human cytomegalovirus IE2 86 kDa protein induces STING degradation and inhibits cGAMP-mediated IFN-β induction. Front. Microbiol. 8:1854. 10.3389/fmicb.2017.01854 29018427 PMC5622937

[B97] Kuriakose T. Man S. M. Subbarao Malireddi R. K. Karki R. Kesavardhana S. Place D. E.. (2016). ZBP1/DAI is an innate sensor of influenza virus triggering the NLRP3 inflammasome and programmed cell death pathways. Sci. Immunol. 1:aag2045. 10.1126/sciimmunol.aag2045 27917412 PMC5131924

[B98] Lahaye X. Satoh T. Gentili M. Cerboni S. Conrad C. Hurbain I.. (2013). The Capsids of HIV-1 and HIV-2 determine immune detection of the viral cdna by the innate sensor cgas in dendritic cells. Immunity 39, 1132–1142. 10.1016/j.immuni.2013.11.002 24269171

[B99] Lee K. G. Kim S. S. Y. Kui L. Voon D. C. C. Mauduit M. Bist P.. (2015). Bruton's tyrosine kinase phosphorylates DDX41 and activates its binding of dsDNA and STING to initiate type 1 interferon response. Cell Rep. 10, 1055–1065. 10.1016/j.celrep.2015.01.039 25704810

[B100] Li X. D. Wu J. Gao D. Wang H. Sun L. Chen Z. J. (2013). Pivotal roles of cGAScGAMP signaling in antiviral defense and immune adjuvant effects. Science 341, 1390–1394. 10.1126/science.1244040 23989956 PMC3863637

[B101] Li Y. Wu Y. Zheng X. Cong J. Liu Y. Li J.. (2016). Cytoplasm-translocated Ku70/80 complex sensing of HBV DNA induces hepatitis-associated chemokine secretion. Front. Immunol. 7:569. 10.3389/fimmu.2016.00569 27994596 PMC5136554

[B102] Lin J. Kumari S. Kim C. Van T. M. Wachsmuth L. Polykratis A.. (2016). RIPK1 counteracts ZBP1-mediated necroptosis to inhibit inflammation. Nature 540, 124–128. 10.1038/nature20558 27819681 PMC5755685

[B103] Lippmann J. Rothenburg S. Deigendesch N. Eitel J. Meixenberger K. van Laak V. (2008). IFNβ responses induced by intracellular bacteria or cytosolic DNA in different human cells do not require ZBP1 (DLM-1/DAI). Cell. Microbiol. 10, 2579–2588. 10.1111/j.1462-5822.2008.01232.x 18771559

[B104] Liu X. Chauhan V. S. Young A. B. Marriott I. (2010). NOD2 mediates inflammatory responses of primary murine glia to *Streptococcus pneumoniae*. Glia 58, 839–847. 10.1002/glia.20968 20091781 PMC2967038

[B105] Lo Cigno I. Calati F. Borgogna C. Zevini A. Albertini S. Martuscelli L.. (2020). Human papillomavirus E7 oncoprotein subverts host innate immunity via SUV39H1-mediated epigenetic silencing of immune sensor genes. J. Virol. 94:19. 10.1128/JVI.01812-19 31776268 PMC6997746

[B106] Lo Cigno I. De Andrea M. Borgogna C. Albertini S. Landini M. M. Peretti A.. (2015). The Nuclear DNA Sensor IFI16 acts as a restriction factor for human papillomavirus replication through epigenetic modifications of the viral promoters. J. Virol. 89, 7506–7520. 10.1128/JVI.00013-15 25972554 PMC4505635

[B107] Luecke S. Holleufer A. Christensen M. H. Jønsson K. L. Boni G. A. Sørensen L. K.. (2017). cGAS is activated by DNA in a length-dependent manner. EMBO Rep. 18, 1707–1715. 10.15252/embr.201744017 28801534 PMC5623850

[B108] Lugrin J. Martinon F. (2018). The AIM2 inflammasome: Sensor of pathogens and cellular perturbations. Immunol. Rev. 281, 99–114. 10.1111/imr.12618 29247998

[B109] Lum K. K. Howard T. R. Pan C. Cristea I. M. (2019). Charge-mediated pyrin oligomerization nucleates antiviral IFI16 sensing of herpesvirus DNA. MBio 10, e01428–19. 10.1128/mBio.01428-19 31337724 PMC6650555

[B110] Lupfer C. Malik A. Kanneganti T. D. (2015). Inflammasome control of viral infection. Curr. Opin. Virol. 12, 38–46. 10.1016/j.coviro2015.02.007 25771504 PMC4470791

[B111] Ma F. Li B. Liu S. Iyer S. S. Yu Y. Wu A.. (2015). Positive feedback regulation of type I IFN production by the IFN-inducible DNA sensor cGAS. J. Immunol. 194, 1545–1554. 10.4049/jimmunol.1402066 25609843 PMC4324085

[B112] Ma J. X. Li J. Y. Fan D. D. Feng W. Lin A. F. Xiang L. X.. (2018). Identification of DEAD-Box RNA helicase DDX41 as a trafficking protein that involves in multiple innate immune signaling pathways in a zebrafish model. Front. Immunol. 9:1327. 10.3389/fimmu.2018.01327 29942316 PMC6005158

[B113] Ma Z. Jacobs S. R. West J. A. Stopford C. Zhang Z. Davis Z.. (2015). Modulation of the cGAS-STING DNA sensing pathway by gammaherpesviruses. Proc. Natl. Acad. Sci. U.S.A. 112, E4306–E4315. 10.1073/pnas.1503831112 26199418 PMC4534226

[B114] Maelfait J. Liverpool L. Bridgeman A. Ragan K. B. Upton J. W. Rehwinkel J. (2017). Sensing of viral and endogenous RNA by ZBP 1/ DAI induces necroptosis. EMBO J. 36, 2529–2543. 10.15252/embj.201796476 28716805 PMC5579359

[B115] Man S. M. Karki R. Kanneganti T. D. (2016). AIM2 inflammasome in infection, cancer, and autoimmunity: role in DNA sensing, inflammation, and innate immunity. Eur. J. Immunol. 46, 269–280. 10.1002/eji.201545839 26626159 PMC4758349

[B116] Maruzuru Y. Ichinohe T. Sato R. Miyake K. Okano T. Suzuki T.. (2018). Herpes Simplex Virus 1 VP22 Inhibits AIM2-dependent inflammasome activation to enable efficient viral replication. Cell Host Microbe 23, 254–265.e7. 10.1016/j.chom.2017.12.014 29447697

[B117] Menendez C. M. Carr D. J. J. (2017). Defining nervous system susceptibility during acute and latent herpes simplex virus-1 infection. J. Neuroimmunol. 308, 43–49. 10.1016/j.jneuroim.2017.02.020 28302316 PMC5474347

[B118] Miao E. A. Rajan J. V. Aderem A. (2011). Caspase-1-induced pyroptotic cell *death*. Immunol. Rev. 243, 206–214. 10.1111/j.1600-065X.2011.01044.x 21884178 PMC3609431

[B119] Moriyama M. Koshiba T. Ichinohe T. (2019). Influenza A virus M2 protein triggers mitochondrial DNA-mediated antiviral immune responses. Nat. Commun. 10:4624. 10.1038/s41467-019-12632-5 31604929 PMC6789137

[B120] Nair S. Diamond M. S. (2015). Innate immune interactions within the central nervous system modulate pathogenesis of viral infections. Curr. Opin. Immunol. 36, 47–53. 10.1016/j.coi.2015.06.011 26163762 PMC4593735

[B121] Nakaya Y. Lilue J. Stavrou S. Moran E. A. Ross S. R. (2017). AIM2-like receptors positively and negatively regulate the interferon response induced by cytosolic DNA. MBio 8:17. 10.1128/mBio.00944-17 28679751 PMC5573678

[B122] Orzalli M. H. Broekema N. M. Diner B. A. Hancks D. C. Elde N. C. Cristea I. M.. (2015). CGAS-mediated stabilization of IFI16 promotes innate signaling during herpes simplex virus infection. Proc. Natl. Acad. Sci. U.S.A. 112, E1773–E1781. 10.1073/pnas.1424637112 25831530 PMC4394261

[B123] Orzalli M. H. Broekema N. M. Knipe D. M. (2016). Relative contributions of herpes simplex virus 1 ICP0 and vhs to loss of cellular IFI16 vary in different human cell types. J. Virol. 90, 8351–8359. 10.1128/JVI.00939-16 27412599 PMC5008076

[B124] Orzalli M. H. DeLuca N. A. Knipe D. M. (2012). Nuclear IFI16 induction of IRF-3 signaling during herpesviral infection and degradation of IFI16 by the viral ICP0 protein. Proc. Natl. Acad. Sci. U.S.A. 109, E3008–E3017. 10.1073/pnas.1211302109 23027953 PMC3497734

[B125] Paijo J. Döring M. Spanier J. Grabski E. Nooruzzaman M. Schmidt T.. (2016). cGAS senses human cytomegalovirus and induces type i interferon responses in human monocyte-derived cells. PLoS Pathog. 12:e1005546. 10.1371/journal.ppat.1005546 27058035 PMC4825940

[B126] Pan S. Liu X. Ma Y. Cao Y. He B. (2018). Herpes simplex virus 1 γ 1 34.5 protein inhibits STING activation that restricts viral replication. J. Virol. 92, e01015–18. 10.1128/JVI.01015-18 30045990 PMC6158424

[B127] Parker Z. M. Murphy A. A. Leib D. A. (2015). Role of the DNA sensor STING in protection from lethal infection following corneal and intracerebral challenge with herpes simplex virus 1. J. Virol. 89, 11080–11091. 10.1128/JVI.00954-15 26311879 PMC4621135

[B128] Parvatiyar K. Zhang Z. Teles R. M. Ouyang S. Jiang Y. Iyer S. S.. (2012). The helicase DDX41 recognizes the bacterial secondary messengers cyclic di-GMP and cyclic di-AMP to activate a type i interferon immune response. Nat. Immunol. 13, 1155–1161. 10.1038/ni.2460 23142775 PMC3501571

[B129] Peters N. E. Ferguson B. J. Mazzon M. Fahy A. S. Krysztofinska E. Arribas-Bosacoma R.. (2013). A Mechanism for the inhibition of DNA-PK-mediated DNA sensing by a Virus. PLoS Pathog. 9:1003649. 10.1371/journal.ppat.1003649 24098118 PMC3789764

[B130] Pham T. H. Kwon K. M. Kim Y. E. Kim K. K. Ahn J. H. (2013). DNA sensing-independent inhibition of herpes simplex virus 1 replication by DAI/ZBP1. J. Virol. 87, 3076–3086. 10.1128/JVI.02860-12 23283962 PMC3592125

[B131] Phelan T. Little M. A. Brady G. (2020). Targeting of the cGAS-STING system by DNA viruses. Biochem. Pharmacol. 174:113831. 10.1016/j.bcp.2020.113831 32004549

[B132] Pisano G. Roy A. Ahmed Ansari M. Kumar B. Chikoti L. Chandran B. (2017). Interferon-γ-inducible protein 16 (IFI16) is required for the maintenance of Epstein-Barr virus latency. Virol. J. 14:221. 10.1186/s12985-017-0891-5 29132393 PMC5683537

[B133] Rathinam V. A. K. Jiang Z. Waggoner S. N. Sharma S. Cole L. E. Waggoner L.. (2010). The AIM2 inflammasome is essential for host defense against cytosolic bacteria and DNA viruses. Nat. Immunol. 11, 395–402. 10.1038/ni.1864 20351692 PMC2887480

[B134] Rebsamen M. Heinz L. X. Meylan E. Michallet M. C. Schroder K. Hofmann K.. (2009). DAI/ZBP1 recruits RIP1 and RIP3 through RIP homotypic interaction motifs to activate NF-κB. EMBO Rep. 10, 916–922. 10.1038/embor.2009.109 19590578 PMC2726668

[B135] Reinert L. S. Lopušná K. Winther H. Sun C. Thomsen M. K. Nandakumar R.. (2016). Sensing of HSV-1 by the cGAS-STING pathway in microglia orchestrates antiviral defence in the CNS. Nat. Commun. 7:13348. 10.1038/ncomms13348 27830700 PMC5109551

[B136] Reinholz M. Kawakami Y. Salzer S. Kreuter A. Dombrowski Y. Koglin S.. (2013). HPV16 activates the AIM2 inflammasome in keratinocytes. Arch. Dermatol. Res. 305, 723–732. 10.1007/s00403-013-1375-0 23764897

[B137] Roos K. L. (1999). Encephalitis. Neurol. Clin. 17, 813–833. 10.1016/S0733-8619(05)70168-7 10517930

[B138] Rothan H. A. Arora K. Natekar J. P. Strate P. G. Brinton M. A. Kumar M. (2019). ZDNA-binding protein 1 is critical for controlling virus replication and survival in west nile virus encephalitis. Front. Microbiol. 10:89. 10.3389/fmicb.2019.02089 31572318 PMC6749019

[B139] Roy A. Dutta D. Iqbal J. Pisano G. Gjyshi O. Ansari M. A.. (2016). Nuclear innate immune DNA sensor IFI16 Is degraded during lytic reactivation of kaposi's sarcomaassociated herpesvirus (KSHV): role of ifi16 in maintenance of kshv latency. J. Virol. 90, 8822–8841. 10.1128/JVI.01003-16 27466416 PMC5021400

[B140] Roy A. Ghosh A. Kumar B. Chandran B. (2019). IfI16, a nuclear innate immune DNA sensor, mediates epigenetic silencing of herpesvirus genomes by its association with H3K9 methyltransferases SUV39H1 and GLP. Elife 8:e49500. 10.7554/eLife.49500 31682228 PMC6855800

[B141] Royer D. J. Carr D. J. J. (2016). A STING-dependent innate-sensing pathway mediates resistance to corneal HSV-1 infection via upregulation of the antiviral effector tetherin. Mucosal Immunol. 9, 1065–1075. 10.1038/mi.2015.124 26627457 PMC4889566

[B142] Sagulenko V. Thygesen S. J. Sester D. P. Idris A. Cridland J. A. Vajjhala P. R.. (2013). AIM2 and NLRP3 inflammasomes activate both apoptotic and pyroptotic death pathways via ASC. Cell Death Differ. 20, 1149–1160. 10.1038/cdd.2013.37 23645208 PMC3741496

[B143] Sawai H. (2016). Induction of apoptosis in TNF-Treated L929 cells in the presence of necrostatin-1. Int. J. Mol. Sci. 17:1678. 10.3390/ijms17101678 27739412 PMC5085711

[B144] Schattgen S. A. Gao G. Kurt-Jones E. A. Fitzgerald K. A. (2016). Cutting edge: dna in the lung microenvironment during influenza virus infection tempers inflammation by engaging the DNA Sensor AIM2. J. Immunol. 196, 29–33. 10.4049/jimmunol.1501048 26590313 PMC4793160

[B145] Schmucker D. Vorbrüggen G. Yeghiayan P. Fan H. Q. Jäckle H. Gaul U. (2000). The Drosophila gene abstrakt, required for visual system development, encodes a putative RNA helicase of the DEAD box protein family. Mech. Dev. 91, 189–196. 10.1016/S0925-4773(99)00298-1 10704843

[B146] Scutts S. R. Ember S. W. Ren H. Ye C. Lovejoy C. A. Mazzon M.. (2018). DNA-PK is targeted by multiple vaccinia virus proteins to inhibit DNA sensing. Cell Rep. 25, 1953–1965.e4. 10.1016/j.celrep.2018.10.034 30428360 PMC6250978

[B147] Semenova N. Bosnjak M. Markelc B. Znidar K. Cemazar M. Heller L. (2019). Multiple cytosolic DNA sensors bind plasmid DNA after transfection. Nucleic Acids Res. 47, 10235–10246. 10.1093/nar/gkz768 31495892 PMC6821305

[B148] Serramía M. J. Muñoz-Fernández M. Á. Álvarez S. (2015). HIV-1 increases TLR responses in human primary astrocytes. Sci. Rep. 5:17887. 10.1038/srep17887 26671458 PMC4680863

[B149] Shi J. Zhao Y. Wang K. Shi X. Wang Y. Huang H.. (2015). Cleavage of GSDMD by inflammatory caspases determines pyroptotic cell death. Nature 526, 660–665. 10.1038/nature15514 26375003

[B150] Shrivastava G. León-Juárez M. García-Cordero J. Meza-Sánchez D. E. Cedillo-Barrón L. (2016). Inflammasomes and its importance in viral infections. Immunol. Res. 64, 1101–1117. 10.1007/s12026-016-8873-z 27699580

[B151] Song X. Ma F. Herrup K. (2019). Accumulation of cytoplasmic DNA Due to ATM deficiency activates the microglial viral response system with neurotoxic consequences. J. Neurosci. 39, 6378–6394. 10.1523/JNEUROSCI.0774-19.2019 31189575 PMC6687895

[B152] Sridharan H. Ragan K. B. Guo H. Gilley R. P. Landsteiner V. J. Kaiser W. J.. (2017). Murine cytomegalovirus IE 3-dependent transcription is required for DAI/ZBP 1mediated necroptosis. EMBO Rep. 18, 1429–1441. 10.15252/embr.201743947 28607035 PMC5538628

[B153] Stabell A. C. Meyerson N. R. Gullberg R. C. Gilchrist A. R. Webb K. J. Old W. M.. (2018). Dengue viruses cleave STING in humans but not in nonhuman primates, their presumed natural reservoir. Elife 7:e31919. 10.7554/eLife.31919 29557779 PMC5860865

[B154] Stavrou S. Aguilera A. N. Blouch K. Ross S. R. (2018). DDX41 recognizes RNA/DNA retroviral reverse transcripts and is critical for *in vivo* control of murine leukemia virus infection. MBio 9, e00923–18. 10.1128/mBio.00923-18 29871919 PMC5989071

[B155] Stavrou S. Blouch K. Kotla S. Bass A. Ross S. R. (2015). Nucleic acid recognition orchestrates the anti-viral response to retroviruses. Cell Host Microbe 17, 478–488. 10.1016/j.chom.2015.02.021 25816774 PMC4393365

[B156] Sterka D. Rati D. M. Marriott I. (2006). Functional expression of NOD2, a novel pattern recognition receptor for bacterial motifs, in primary murine astrocytes. Glia 53, 322–330. 10.1002/glia.20286 16265673

[B157] Su C. Zheng C. (2017). Herpes simplex virus 1 abrogates the cGAS/STING-mediated cytosolic DNA-sensing pathway via its virion host shutoff protein, UL41. J. Virol. 91, e02414–16. 10.1128/JVI.02414-16 28077645 PMC5331819

[B158] Su J. Rui Y. Lou M. Yin L. Xiong H. Zhou Z.. (2019). HIV-2/SIV Vpx targets a novel functional domain of STING to selectively inhibit cGAS–STING-mediated NF-κB signalling. Nat. Microbiol. 4, 2552–2564. 10.1038/s41564-019-0585-4 31659299

[B159] Sui H. Zhou M. Imamichi H. Jiao X. Sherman B. T. Clifford Lane H.. (2017). STING is an essential mediator of the Ku70-mediated production of IFN-γ1 in response to exogenous DNA. Sci. Signal. 10:aah5054. 10.1126/scisignal.aah5054 28720717

[B160] Sun B. Sundström K. B. Chew J. J. Bist P. Gan E. S. Tan H. C.. (2017). Dengue virus activates cGAS through the release of mitochondrial DNA. Sci. Rep. 7:3594. 10.1038/s41598-017-03932-1 28620207 PMC5472572

[B161] Sun L. Wu J. Du F. Chen X. Chen Z. J. (2013). Cyclic GMP-AMP synthase is a cytosolic DNA sensor that activates the type I interferon pathway. Science 39, 786–91. 10.1126/science.1232458 23258413 PMC3863629

[B162] Sun L. Xing Y. Chen X. Zheng Y. Yang Y. Nichols D. B.. (2012). Coronavirus papain-like proteases negatively regulate antiviral innate immune response through disruption of STING-mediated signaling. PLoS ONE 7:30802. 10.1371/journal.pone.0030802 22312431 PMC3270028

[B163] Swanson K. V. Junkins R. D. Kurkjian C. J. Holley-Guthrie E. Pendse A. A. Morabiti R.. (2017). A noncanonical function of cGAMP in inflammasome priming and activation. J. Exp. Med. 214, 3611–3262. 10.1084/jem.20171749 29030458 PMC5716045

[B164] Takaoka A. Wang Z. Choi M. K. Yanai H. Negishi H. Ban T.. (2007). DAI (DLM1/ZBP1) is a cytosolic DNA sensor and an activator of innate immune response. Nature 448, 501–505. 10.1038/nature06013 17618271

[B165] Thapa R. J. Ingram J. P. Ragan K. B. Nogusa S. Boyd D. F. Benitez A. A.. (2016). DAI senses influenza a virus genomic RNA and activates RIPK3-dependent cell death. Cell Host Microbe 20, 674–681. 10.1016/j.chom.2016.09.014 27746097 PMC5687825

[B166] Unterholzner L. (2013). The interferon response to intracellular DNA: why so many receptors? Immunobiology 218, 1312–1321. 10.1016/j.imbio.2013.07.007 23962476

[B167] Unterholzner L. Keating S. E. Baran M. Horan K. A. Jensen S. B. Sharma S.. (2010). IFI16 is an innate immune sensor for intracellular DNA. Nat. Immunol. 11, 997–1004. 10.1038/ni.1932 20890285 PMC3142795

[B168] Upton J. W. Kaiser W. J. Mocarski E. S. (2012). DAI/ZBP1/DLM-1 Complexes with RIP3 to mediate virus-induced programmed necrosis that is targeted by murine cytomegalovirus vIRA. Cell Host Microbe 11, 290–297. 10.1016/j.chom.2012.01.016 22423968 PMC3531981

[B169] Vemuri M. C. Schiller E. Naegele J. R. (2001). Elevated DNA double strand breaks and apoptosis in the CNS of scid mutant mice. Cell Death Differ. 8, 245–255. 10.1038/sj.cdd.4400806 11319607

[B170] Venkatesan A. Geocadin R. G. (2014). Diagnosis and management of acute encephalitis: a practical approach. Neurol. Clin. Pract. 4, 206–215. 10.1212/CPJ.0000000000000036 25110619 PMC4121461

[B171] Vermeire J. Roesch F. Sauter D. Rua R. Hotter D. Van Nuffel A.. (2016). HIV Triggers a cGAS-dependent, Vpu- and Vpr-regulated type i interferon response in CD4+ T Cells. Cell Rep. 17, 413–424. 10.1016/j.celrep.2016.09.023 27705790

[B172] Wang J. Kang L. Song D. Liu L. Yang S. Ma L.. (2017). Ku70 Senses HTLV-1 DNA and modulates HTLV-1 replication. J. Immunol. 199, 2475–2482. 10.4049/jimmunol.1700111 28821586

[B173] Wang X. Li Y. Liu S. Yu X. Li L. Shi C.. (2014). Direct activation of RIP3/MLKLdependent necrosis by herpes simplex virus 1 (HSV-1) protein ICP6 triggers host antiviral defense. Proc. Natl. Acad. Sci. U.S.A. 111, 15438–15443. 10.1073/pnas.1412767111 25316792 PMC4217423

[B174] Wang Z. C. Choi M. K. Ban T. Yanai H. Negishi H. Lu Y.. (2008). Regulation of innate immune responses by DAI (DLM-1/ZBP1) and other DNA-sensing molecules. Proc. Natl. Acad. Sci. U.S.A. 105, 5477–5482. 10.1073/pnas.0801295105 18375758 PMC2291118

[B175] West A. P. Khoury-Hanold W. Staron M. Tal M. C. Pineda C. M. Lang S. M.. (2015). Mitochondrial DNA stress primes the antiviral innate immune response. Nature 520, 553–557. 10.1038/nature14156 25642965 PMC4409480

[B176] Wong E. B. Montoya B. Ferez M. Stotesbury C. Sigal L. J. (2019). Resistance to ectromelia virus infection requires cGAS in bone marrow-derived cells which can be bypassed with cGAMP therapy. PLoS Pathog. 15:e1008239. 10.1371/journal.ppat.1008239 31877196 PMC6974301

[B177] Wu J. J. Li W. Shao Y. Avey D. Fu B. Gillen J.. (2015). Inhibition of cGAS DNA sensing by a herpesvirus virion protein. Cell Host Microbe 18, 333–344. 10.1016/j.chom.2015.07.015 26320998 PMC4567405

[B178] Xu H. Su C. Pearson A. Mody C. H. Zheng C. (2017). Herpes simplex virus 1 UL24 abrogates the DNA sensing signal pathway by inhibiting NF-κB activation. J. Virol. 91, e00025–17. 10.1128/JVI.00025-17 28100608 PMC5355614

[B179] Yang Y. Zhao X. Wang Z. Shu W. Li L. Li Y.. (2020). Nuclear sensor interferon-inducible protein 16 inhibits the function of hepatitis b virus covalently closed circular DNA by integrating innate immune activation and epigenetic suppression. Hepatology 71, 1154–1169. 10.1002/hep.30897 31402464

[B180] Ye R. Su C. Xu H. Zheng C. (2017). Herpes simplex virus 1 ubiquitin-specific protease ul36 abrogates nf-κb activation in dna sensing signal pathway. J. Virol. 91:e02417–16. 10.1128/JVI.02417-16 28031360 PMC5309955

[B181] Yi G. Wen Y. Shu C. Han Q. Konan K. V. Li P.. (2016). Hepatitis C virus NS4B can suppress STING accumulation to evade innate immune responses. J. Virol. 90, 254–265. 10.1128/JVI.01720-15 26468527 PMC4702547

[B182] Yogarajah T. Ong K. C. Perera D. Wong K. T. (2017). AIM2 Inflammasome-mediated pyroptosis in enterovirus A71-infected neuronal cells restricts viral replication. Sci. Rep. 7:5845. 10.1038/s41598-017-05589-2 28724943 PMC5517550

[B183] You H. Zheng S. Huang Z. Lin Y. Shen Q. Zheng C. (2019). Herpes simplex virus 1 tegument protein ul46 inhibits tank-binding kinase 1-mediated signaling. MBio 10, 919–938. 10.1128/mBio.00919-19 31113902 PMC6529639

[B184] Zhang D. Su C. Zheng C. (2016). Herpes simplex virus 1 serine protease VP24 blocks the dna-sensing signal pathway by abrogating activation of interferon regulatory factor 3. J. Virol. 90, 5824–5829. 10.1128/JVI.00186-16 27076640 PMC4886774

[B185] Zhang G. Chan B. Samarina N. Abere B. Weidner-Glunde M. Buch A.. (2016). Cytoplasmic isoforms of Kaposi sarcoma herpesvirus LANA recruit and antagonize the innate immune DNA sensor cGAS. Proc. Natl. Acad. Sci. U.S.A. 113, E1034–E1043. 10.1073/pnas.1516812113 26811480 PMC4776510

[B186] Zhang H. Luo J. Alcorn J. F. Chen K. Fan S. Pilewski J.. (2017). AIM2 inflammasome is critical for influenza-induced lung injury and mortality. J. Immunol. 198, 4383–4393. 10.4049/jimmunol.1600714 28424239 PMC5439025

[B187] Zhang J. Zhao J. Xu S. Li J. He S. Zeng Y.. (2018). Species-specific deamidation of cGAS by herpes simplex virus UL37 protein facilitates viral replication. Cell Host Microbe 24, 234–248.e5. 10.1016/j.chom.2018.07.004 30092200 PMC6094942

[B188] Zhang X. Brann T. W. Zhou M. Yang J. Oguariri R. M. Lidie K. B.. (2011). Cutting Edge: Ku70 is a novel cytosolic DNA sensor that induces type III rather than type I IFN. J. Immunol. 186, 4541–4545. 10.4049/jimmunol.1003389 21398614 PMC3720676

[B189] Zhang X. Shi H. Wu J. Zhang X. Sun L. Chen C.. (2013). Cyclic GMP-AMP containing mixed phosphodiester linkages is an endogenous high-affinity ligand for STING. Mol. Cell 51, 226–235. 10.1016/j.molcel.2013.05.022 23747010 PMC3808999

[B190] Zhang Z. Bao M. Lu N. Weng L. Yuan B. Liu Y. J. (2013). The E3 ubiquitin ligase TRIM21 negatively regulates the innate immune response to intracellular double-stranded DNA. Nat. Immunol. 14, 172–178. 10.1038/ni.2492 23222971 PMC3645272

[B191] Zhang Z. Yuan B. Bao M. Lu N. Kim T. Liu Y. J. (2011). The helicase DDX41 senses intracellular DNA mediated by the adaptor STING in dendritic cells. Nat. Immunol. 12, 959–965. 10.1038/ni.2091 21892174 PMC3671854

[B192] Zheng Y. Liu Q. Wu Y. Ma L. Zhang Z. Liu T.. (2018). Zika virus elicits inflammation to evade antiviral response by cleaving cGAS via NS1-caspase-1 axis. EMBO J. 37:e99347. 10.15252/embj.201899347 30065070 PMC6138430

[B193] Zhu W. Zu X. Liu S. Zhang H. (2019). The absent in melanoma 2 (AIM2) inflammasome in microbial infection. Clin. Chim. Acta 495, 100–108. 10.1016/j.cca.2019.04.052 30959045

[B194] Zohaib A. Sarfraz A. Kaleem Q. M. Ye J. Mughal M. N. Navid M. T.. (2016). The Yin and Yang of antiviral innate immunity in central nervous system. Curr. Pharm. Des. 22, 648–655. 10.2174/1381612822666151204001550 26635264

